# Topology-driven energy transfer networks for upconversion stimulated emission depletion microscopy

**DOI:** 10.1038/s41377-025-02054-y

**Published:** 2025-12-04

**Authors:** Weizhao Gu, Simone Lamon, Haoyi Yu, Qiming Zhang, Min Gu

**Affiliations:** 1 School of Artificial Intelligence Science and Technology, University of Shanghai for Science and Technology, Shanghai, 200093 China; 2 Institute of Photonic Chips, University of Shanghai for Science and Technology, Shanghai, 200093 China

**Keywords:** Imaging and sensing, Nanoparticles

## Abstract

Lanthanide-doped upconversion nanoparticles enable upconversion stimulated emission depletion microscopy with high photostability and low-intensity near-infrared continuous-wave lasers. Controlling energy transfer dynamics in these nanoparticles is crucial for super-resolution microscopy with minimal laser intensities and high photon budgets. However, traditional methods neglect the spatial distribution of lanthanide ions and its effect on energy transfer dynamics. Here, we introduce topology-driven energy transfer networks in lanthanide-doped upconversion nanoparticles for upconversion stimulated emission depletion microscopy with reduced laser intensities, maintaining a high photon budget. Spatial separation of Yb^3+^ sensitizers and Tm^3+^ emitters in 50-nm core-shell nanoparticles enhance energy transfer dynamics for super-resolution microscopy. Topology-dependent energy migration produces strong 450-nm upconversion luminescence under low-power 980-nm excitation. Enhanced cross-relaxation improves optical switching efficiency, achieving a saturation intensity of 0.06 MW cm^−2^ under excitation at 980 nm and depletion at 808 nm. Super-resolution imaging with a 65-nm lateral resolution is achieved using intensities of 0.03 MW cm^−2^ for a Gaussian-shaped excitation laser at 980 nm and 1 MW cm^−2^ for a donut-shaped depletion laser at 808 nm, representing a 10-fold reduction in excitation intensity and a 3-fold reduction in depletion intensity compared to conventional methods. These findings demonstrate the potential of harnessing topology-dependent energy transfer dynamics in upconversion nanoparticles for advancing low-power super-resolution applications.

## Abstract (web summary)

Topology-engineered upconversion nanoparticles enable low-power STED microscopy by optimizing energy transfer and cross-relaxation, achieving sub-diffraction resolution with significantly reduced excitation and depletion intensities for efficient super-resolution imaging.

## Graphical abstract

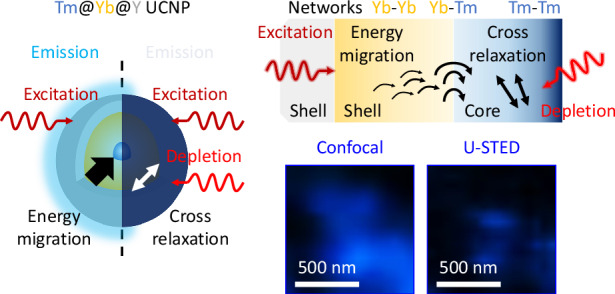

## Introduction

Stimulated emission depletion (STED) microscopy is a far-field super-resolution technique enabling nanoscale 3D imaging, widely applied in life sciences and nanophotonics^1–3^. It employs two overlapping lasers: a Gaussian-shaped laser to excite fluorophores and a donut-shaped laser to suppress fluorescence through stimulated emission, effectively confining emission to the center of the donut. By toggling fluorophores between “ON” and “OFF” states and fine-tuning laser intensities, STED microscopy achieves remarkable spatial resolution^4,5^. Recent advances in STED microscopy have achieved Ångström-level molecular localization with minimal photobleaching through advanced beam control and fluorophore switching^6,7^. Adaptive optics has extended these capabilities to deep 3D imaging with sub-50nm isotropic resolution in thick, aberrating tissues^8^. Integration with deep learning now enables isotropic super-resolution and synapse-level 3D reconstruction of living brain tissue^9^. Additionally, STED-image scanning microscopy reduces depletion intensity and background for gentle imaging of live, thick samples^10^, while event-triggered STED enables rapid, real-time capture of dynamic cellular events^11^. Despite its advantages and recent advancements in optics, computational methods, and probe design^12–14^, challenges such as limited probe photostability and reliance on high-intensity pulsed lasers hinder its broader application. Lanthanide-doped upconversion nanoparticles (UCNPs)^15,16^, characterized by excited states with long lifetime, provide a promising platform for upconversion STED (U-STED) microscopy^17,18^, enabling stable upconversion luminescence (UCL) emission with low-intensity near-infrared (NIR) continuous-wave (CW) lasers.

Precise control of energy transfer dynamics in UCNPs^19^, which stem from complex photophysical transitions among lanthanide ions, is vital for enabling U-STED microscopy with minimal laser intensities and a high photon budget^20^. In Yb^3+^, Tm^3+^-doped UCNPs, increasing Tm^3+^ doping enhances upconversion and optical switching efficiency by shortening inter-emitter distances and promoting cross-relaxation (CR), a critical mechanism for population inversion^21,22^. This strategy reduces depletion laser intensity for U-STED microscopy by two orders of magnitude compared to traditional STED microscopy with fluorophores^23,24^. Additionally, the dynamic response of CR to excitation power further minimizes the required laser intensity^25^. Increasing Yb^3+^ doping boosts UCL emission and accelerates kinetics due to a concentrating effect^26^. However, excessive doping risks concentration quenching^27^. Similarly, raising excitation power increases the photon budget but elevates laser intensity^28,29^. Efficient optical switching is alternatively achievable through distinct upconversion pathways in Yb^3+^, Er^3+^-doped UCNPs with low lanthanide doping^30^, multi-step STED processes in Yb^3+^,Tm^3+^ systems with low lanthanide doping^31^, or a cascade-amplified depletion process targeting shared sensitizers in multichromatic UCNPs^32^. However, traditional methods focus on localized interactions between sensitizers and emitters via lanthanide combinations and concentrations, often neglecting the spatial distribution of lanthanide ions within UCNPs and its impact on energy transfer dynamics.

Recent advancements in UCNP architecture engineering enable precise control of energy transfer dynamics by leveraging distributed interactions between distal lanthanide ions through spatial adjustments within UCNPs, resulting in tunable and enhanced UCL emission^33–37^. These innovations support high-efficiency optical switching for super-resolution microscopy via surface migration-driven emission depletion^38^. Additionally, segregating sensitizers and emitters in core-shell UCNPs enhances UCL emission intensity through topology-dependent energy migration (EM) toward the core-shell interface^39^.

Here, we introduce a novel approach to topology-driven energy transfer networks (ETNs) within lanthanide-doped UCNPs for U-STED microscopy, enabling reduced laser intensities while maintaining a high photon budget. Our theoretical and experimental work shows that spatial separation of Yb^3+^ sensitizers and Tm^3+^ emitters within a 50-nm core-shell UCNP structure facilitates the formation of topology-driven ETNs that channel energy toward the core-shell interface, enhancing dynamics for U-STED microscopy. This approach leverages topology-dependent EM within the Yb^3+^-Yb^3+^ ETN to enhance energy transfer upconversion (ETU) within the Yb^3+^-Tm^3+^ ETN, resulting in strong 450-nm UCL emission under excitation at 980 nm with an intensity of 0.03 MW cm^−2^. Furthermore, this strategy boosts CR in the intermediate excited state of the Tm^3+^-Tm^3+^ ETN, promoting population inversion and improving optical switching efficiency. We achieve a saturation intensity of 0.06 MW cm^−2^ under excitation at 980 nm and depletion at 808 nm. We perform super-resolution imaging with a lateral resolution of 65 nm using intensities of 0.03 MW cm^−2^ for a Gaussian-shaped excitation laser at 980 nm and 1 MW cm^−2^ for a donut-shaped depletion laser at 808 nm. This design achieves a 10-fold reduction in excitation laser intensity and a 3-fold reduction in depletion laser intensity compared to state-of-the-art U-STED microscopy approaches^21,22,25,26^ (Table S1). These findings demonstrate the potential of exploiting energy transfer dynamics dependent on the topology of UCNPs to advance low-power super-resolution applications in life sciences and nanophotonics.

## Results

We developed a dual-beam optical system to investigate the impact of lanthanide ion spatial distribution in individual Yb,Tm-doped core-shell UCNPs with high lanthanide ion doping for U-STED microscopy, minimizing laser intensities while preserving a high photon budget (Fig. S1). We fabricated UCNPs with various core-shell structures: sensitizer+emitter@inert with thin core Yb,Tm@Y and thick core **Yb,Tm**@Y (a typical UCNP structure), sensitizer@emitter@inert with thin intermediate shell Yb@Tm@Y and thick intermediate shell Yb@**Tm**@Y, and emitter@sensitizer@inert with thin intermediate shell Tm@Yb@Y and thick intermediate shell Tm@**Yb**@Y configurations (Fig. S2).

**Fig. 1:**
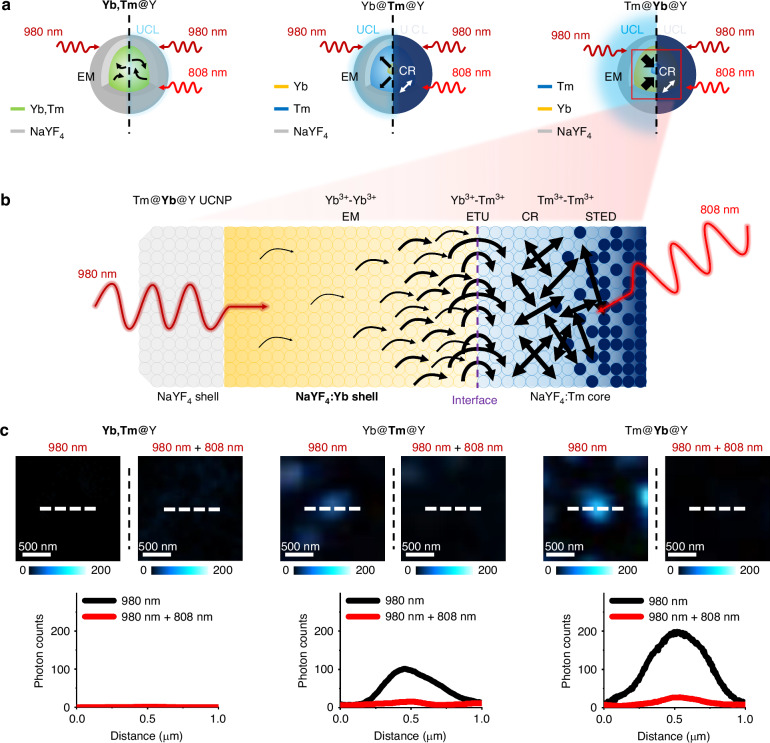
**Probing UCL emission driven by topology-based ETNs in core-shell UCNPs using dual-beam irradiation for U-STED microscopy.** **a** Schematic of 450-nm UCL emission from **Yb,Tm**@Y, Yb@**Tm**@Y, and Tm@**Yb**@Y UCNPs under (left) irradiation with a CW laser at 980 nm and (right) dual-beam irradiation with CW lasers at 980 nm and 808 nm. **b** Illustration of the spatial separation of Yb^3+^ sensitizers and Tm^3+^ emitters within the Tm@**Yb**@Y UCNP structure, highlighting the formation of topology-driven ETNs. These networks channel energy toward the core-shell interface, enhancing energy dynamics for U-STED microscopy. **c** Confocal microscopy images showing 450-nm UCL emission from **Yb,Tm**@Y, Yb@**Tm**@Y, and Tm@**Yb**@Y UCNPs under (left) irradiation with a CW laser at 980 nm and (right) dual-becam irradiation with CW lasers at 980 nm and 808 nm. The UCL emission intensity profile across a single UCNP, measured along the dashed white line, is presented. The intensities of the CW lasers at 980 nm and 808 nm are 0.03 MW cm^−2^ and 1 MW cm^−2^, respectively. Pixel dwell time: 10 ms. Scale bar: 500 nm

Figure 1 illustrates the probing of UCL emission mediated by topology-driven ETNs in core-shell UCNPs under dual-beam irradiation for U-STED microscopy. Figure 1a shows schematics of 450-nm UCL emission from **Yb,Tm**@Y, Yb@**Tm**@Y, and Tm@**Yb**@Y UCNPs under two irradiation conditions: (left) low-power 980-nm CW irradiation and (right) low-power dual-beam irradiation with both 980-nm and 808-nm CW lasers. Under low-power 980-nm CW irradiation, typical **Yb,Tm**@Y UCNPs do not emit 450-nm UCL emission due to inefficient ETU via local energy transfer from Yb^3+^ to Tm^3+^ within the same core. However, low-power dual-beam irradiation with both 980-nm and 808-nm CW lasers results in weak 450-nm UCL emission, attributed to enhanced upconversion via direct 808-nm photon absorption by Tm^3+^. The UCL emission behavior in core-shell UCNPs with topology-driven ETNs differs from conventional systems. In Yb@**Tm**@Y UCNPs, Yb^3+^-Yb^3+^ networks in the core enhance light absorption under low-power 980-nm CW irradiation, but inefficient topology-driven ETU in the Yb^3+^-Tm^3+^ network limits 450-nm UCL emission. Under low-power dual-beam irradiation, Tm^3+^-Tm^3+^ networks in the intermediate shell facilitate intense CR processes, inducing population inversion in the intermediate excited state of Tm^3+^ and enabling efficient optical switching of 450-nm UCL emission. In Tm@**Yb**@Y UCNPs, Yb^3+^-Yb^3+^ networks in the intermediate shell enhance light absorption under low-power 980-nm CW irradiation, and efficient topology-driven ETU in the Yb^3+^-Tm^3+^ network results in strong 450-nm UCL emission. Additionally, low-power dual-beam irradiation promotes intense CR processes in Tm^3+^-Tm^3+^ networks in the intermediate shell, leading to population inversion and efficient optical switching of 450-nm UCL emission.

Figure 1b depicts the spatial separation of Yb^3+^ sensitizers and Tm^3+^ emitters within the Tm@**Yb**@Y UCNP structure. The formation of ETNs^40^ is facilitated by the unique topology of these UCNPs^39^, channeling energy efficiently toward the core-shell interface, which enhances energy dynamics critical for U-STED microscopy, according to the proposed mechanism as follows. The Yb^3+^-Yb^3+^ networks in the intermediate shell strongly absorb 980-nm excitation photons^15,16^, driving topology-mediated EM toward the core-shell interface^39–42^. The Yb^3+^-Tm^3+^ networks at the interface induce efficient topology-driven ETU, exciting Tm^3+^ in the core and generating 450-nm UCL emission^19,20^. The Tm^3+^-Tm^3+^ networks within the core promote CR pathways, leading to population inversion in the intermediate Tm^3+^ excited state and enabling efficient optical switching of 450-nm UCL emission through STED under 808-nm depletion laser irradiation^21,22,25,26^. Evidence and arguments supporting the proposed mechanism are provided in the following sections.

We tested the three UCNP configurations under two irradiation conditions. Figure 1c presents confocal microscopy images of the 450-nm UCL emission from the UCNPs, captured under (left) low-power CW Gaussian-shaped excitation laser at 980 nm alone and (right) low-power dual-beam irradiation with an additional CW Gaussian-shaped depletion laser at 808 nm. A corresponding UCL emission intensity profile along the dashed white line illustrates the spatial resolution and intensity of 450-nm UCL emission under each condition. The CW excitation and depletion lasers at 980 nm and 808 nm were operated at intensities of 0.03 MW cm^−2^ and 1 MW cm^−2^, respectively, with a pixel dwell time of 10 ms. No 450-nm UCL emission is observed from typical **Yb,Tm**@Y UCNPs under low-power 980-nm CW irradiation, but weak 450-nm UCL emission is detected under low-power dual-beam irradiation with both 980-nm CW and 808-nm CW lasers. In Yb@**Tm**@Y UCNPs, 450-nm UCL emission is detected under low-power 980-nm CW irradiation, and it is suppressed under dual-beam 980-nm CW and 808-nm CW irradiation. In Tm@**Yb**@Y UCNPs, strong 450-nm UCL emission occurs under low-power 980-nm CW irradiation, and it is suppressed under low-power dual-beam 980-nm CW and 808-nm CW irradiation. These results demonstrate that our novel core-shell UCNP structure, which leverages topology-driven ETNs under dual-beam irradiation, optimizes energy interactions between lanthanide ions through a carefully engineered spatial distribution within the nanoparticles. This design enables lower laser intensities while maintaining a high photon budget, highlighting its significant potential for U-STED microscopy.

To investigate the relationship between the spatial distribution of lanthanide ions within UCNPs and the enhancement of U-STED microscopy performance, we design and characterize core-shell UCNPs driven by topology-based ETNs. In these designs, Yb^3+^ ions are used as sensitizers and Tm^3+^ ions as emitters, both doped at high concentrations. In the first structure (NaYF₄:25%Yb,5%Tm@NaYF₄ UCNPs), Yb^3+^ and Tm^3+^ are intermixed within a single core to facilitate local energy transfer. In the “core-to-shell” (NaYF₄:25%Yb@5%Tm@NaYF₄ UCNPs) and “shell-to-core” (NaYF₄:5%Tm@25%Yb@NaYF₄ UCNPs) configurations, Yb^3+^ forms the spherical core with Tm^3+^ in the shell, or vice versa. The core and shell volumes are kept equal, allowing for a direct comparison of energy transfer efficiencies between the two compartments (Table S2). The terms “core-to-shell” and “shell-to-core” indicate the direction of energy transfer, either from the core (sensitizer) to the shell (emitter) or in reverse. To minimize surface quenching effects and enhance upconversion efficiency, all three configurations include an outermost inert NaYF₄ shell. Structural analysis of the core-shell UCNPs is conducted using transmission electron microscopy (TEM), which reveals their morphology. The core-shell UCNPs with thin cores and intermediate shells have a size of approximately 30 nm, while the core-shell UCNPs with thick cores and intermediate shells measure around 50 nm, both exhibiting a homogeneous size distribution (Fig. S3). X-ray diffraction analysis further confirms their hexagonal *β*-NaYF₄ phase (Fig. S4).

**Fig. 2:**
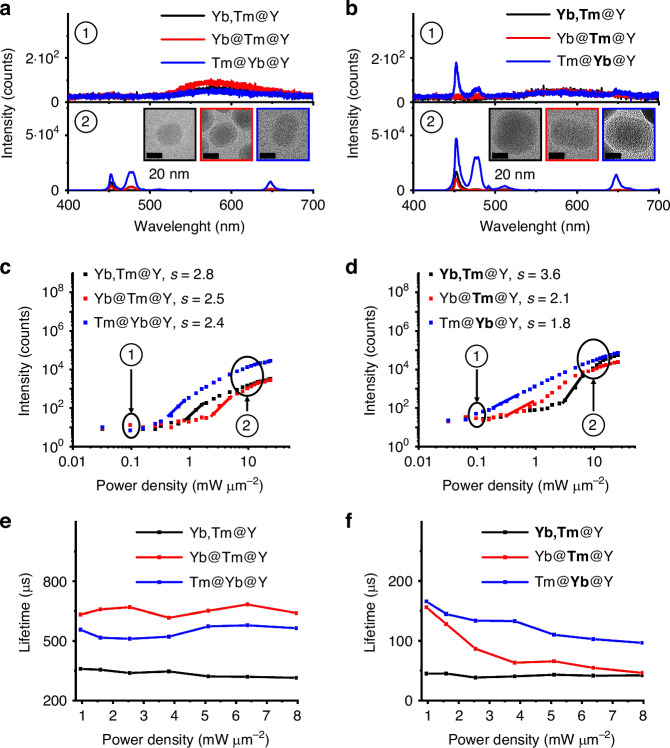
**Characterization of UCL emission driven by topology-based ETNs in core-shell UCNPs for U-STED microscopy.** **a** UCL emission spectra of core-shell UCNPs with thin cores and intermediate shells under CW laser excitation at 980 nm with power densities of 0.1 mW μm^−2^ (top) and 10 mW μm^−2^ (bottom). Exposure time: 2 s. Inset: high-resolution TEM images of the nanoparticles. Scale bar: 20 nm. **b** Same to (**a**), but for core-shell UCNPs with thick cores and intermediate shells. **c** Power-dependent 450-nm UCL emission spectra of core-shell UCNPs with thin cores and intermediate shells under CW laser excitation at 980 nm with increasing power densities. **d** Same to (**c**), but for core-shell UCNPs with thick cores and intermediate shells. **e** Power-dependent 450-nm UCL emission lifetime measurements for core-shell UCNPs with thin cores and intermediate shells under 980-nm CW laser excitation. **f** Same to (**e**), but for core-shell UCNPs with thick cores and intermediate shells

Figure 2 presents the characterization of UCL emission mediated by topology-based ETNs in core-shell UCNPs for U-STED microscopy under CW excitation at 980 nm. We compare the UCL emission spectra of UCNPs with thin cores and intermediate shells (Fig. 2a) and thick cores and intermediate shells (Fig. 2b) under CW laser excitation at 980 nm with power densities of 0.1 mW μm^−2^ (top, indicated as ①) and 10 mW μm^−2^ (bottom, indicated as ②). The proposed UCL emission mechanism involves the absorption of photons at 980 nm by Yb^3+^ ions, which then transfer energy to Tm^3+^ ions via ETU, facilitating upconversion from the ^3^H_6_ ground state to the ^1^D_2_ excited state of Tm^3+^. The UCL emission at 450 nm originates from the ^1^D_2_ to ^3^F_4_ transition, with additional emissions at 475 nm, 650 nm, and 808 nm (not shown), corresponding to the ^1^G_4_ to ^3^H_6_, ^1^G_4_ to ^3^F_4_, and ^3^H_4_ to ^3^H_6_ transitions of Tm^3+^, respectively (Fig. S5). Insets in both panels display high-resolution TEM images of the nanoparticles, illustrating their structural morphology.

For 30-nm UCNPs, no UCL emission is observed under 0.1 mW μm^−2^ irradiation, but UCL emission appears at 10 mW μm^−2^, with the Tm@Yb@Y UCNPs showing the brightest UCL emission. In 50-nm UCNPs, UCL emission is detected at both 0.1 mW μm^−2^ and 10 mW μm^−2^, with Tm@**Yb**@Y UCNPs again exhibiting the brightest emission. These results indicate that the topological arrangement in core-shell UCNPs with ETNs enhances UCL emission via topology-dependent EM from Yb^3+^ sensitizers to Tm^3+^ emitters. This process favors UCL emission in the shell-to-core structure, leading to over an order of magnitude increase in UCL emission brightness compared to typical core-shell UCNPs. However, this enhancement is only observed in larger nanoparticles, suggesting efficient activation of ETNs for improved light absorption under low-power 980-nm CW irradiation^43^.

We illustrate the power-dependent 450-nm UCL emission spectra for core-shell UCNPs with thin cores or intermediate shells (Fig. 2c) and thick cores or intermediate shells (Fig. 2d) under increasing power densities of the CW laser excitation at 980 nm. The intensity of UCL emission *I*_*UCL*_ typically follows a power-law relationship with excitation power *P*, expressed as *I*_*UCL*_∝*P*^*n*^, where the exponent *n* represents the number of photons involved in the multiphoton absorption process^44^. For the 450-nm UCL emission attributed to the transition from ^1^D_2_ to ^3^F_4_ of the Tm^3+^ ions, this transition is typically described as a process involving three or four photons^45,46^. For core-shell UCNPs that do not exhibit topology-driven ETN behavior, the power-dependent 450-nm UCL emission measurement confirms conventional upconversion processes involving three photons, with slopes of 2.8 for Yb,Tm@Y, 2.5 for Yb@Tm@Y, and 2.4 for Tm@Yb@Y UCNPs. Similarly, larger core-shell UCNPs without topology-driven ETN behavior show a slope of 3.6 for **Yb,Tm**@Y UCNPs, corresponding to a four-photon upconversion process. In contrast, core-shell UCNPs exhibiting topology-driven ETN behavior display unconventional upconversion processes involving two photons. This is reflected in reduced slopes of 2.1 for Yb@**Tm**@Y and 1.8 for Tm@**Yb**@Y UCNPs. This deviation is attributed to the efficient ET mechanism in the shell-to-core UCNP structure, which minimizes back energy transfer by separating Yb^3+^ and Tm^3+^ ions^39^. This design enhances the population of excited Tm^3+^ intermediates and exhibits greater robustness to power density variations compared to core-to-shell structures. These findings underscore the importance of topology-driven ETNs in modulating UCL emission dynamics and offer valuable insights into the energy dynamics of upconversion processes for U-STED microscopy.

Understanding the origin of the 450-nm UCL emission behavior requires exploring the topology-driven formation of ETNs in core-shell UCNPs. To investigate this, we analyze the excited-state dynamics—key features of these complex systems—using time-resolved photoluminescence measurements. We report the power-dependent 450-nm UCL emission lifetimes for core-shell UCNPs with thin (Fig. 2e) or thick (Fig. 2f) cores and intermediate shells under 980-nm CW laser excitation, where lifetime is the time for an intensity drop by 1/e. Using time-resolved luminescence, we investigate the origins of ET dynamics and luminescence lifetimes in these UCNP structures. We find that the lifetimes of 450-nm UCL emission from core-shell UCNPs with thick cores or intermediate shells (44.8 µs for **Yb,Tm**@Y, 155.8 µs for Yb@**Tm**@Y, and 165.6 µs for Tm@**Yb**@Y UCNPs) under excitation at 980 nm with a power density of 1 mW μm^−2^ are shorter than those with thin cores or intermediate shells (358.6 µs for Yb,Tm@Y, 632.1 µs for Yb@Tm@Y, and 556.2 µs for Tm@Yb@Y UCNPs under excitation at 980 nm with a power density of 1 mW μm^−2^. This reduction is attributed to accelerated inter-ionic dynamics in larger nanoparticles, driven by the higher concentration of doped lanthanide ions^21,22,25,26^.

For 50-nm core-shell UCNPs with topology-dependent ETN behavior, the lifetimes of UCNPs with shell-to-core topology-driven dynamics are longer than those with local or core-to-shell topology-driven dynamics. The longer lifetime in the shell-to-core configuration may result from the interface that minimizes the quenching effect of back energy transfer from Tm^3+^ to Yb^3+^. In contrast, the reduced lifetime in the core-to-shell architecture likely arises from the proximity of Tm^3+^ ions to surface quenchers in the interior shell. Similar trend between luminescence and lifetime was observed in other lanthanide-based UCNP systems, where longer luminescent lifetimes typically indicate stronger emission intensity^47–50^. Furthermore, our results show that the luminescence lifetimes of 450-nm UCNPs exhibiting topology-driven ETNs decrease with increasing excitation power density, from 165.6 µs under excitation at 980 nm with a power density of 1 mW μm^−2^ to 96.5 µs under excitation at 980 nm with a power density of 8 mW μm^−2^. This behavior is attributed to the intrinsic relaxation rates of individual transitions, with the lifetime data interpreted by considering each core-shell UCNP as a complex ET network^40^. These power-dependent lifetimes reflect the collective effect of multiple minor relaxation pathways that depopulate and repopulate the Tm^3+^ emitting levels (Fig. S6). At higher excitation powers, Tm^3+^ states are dynamically modulated through the cumulative effect of multiple, seemingly minor ET processes rather than a single dominant route. This networked interaction leads to nonlinear changes in lifetime behavior, driven by feedback mechanisms—both positive and negative—within the ETN. Such complexity underpins the power-sensitive dynamics of UCNP luminescence and provides mechanistic insight into the lifetime variations observed. Our findings highlight the critical role of topology-driven ETNs in influencing UCL emission dynamics, providing valuable insights into the mechanisms governing upconversion behavior in core-shell UCNPs. These insights are crucial for optimizing their performance in U-STED microscopy.

**Fig. 3:**
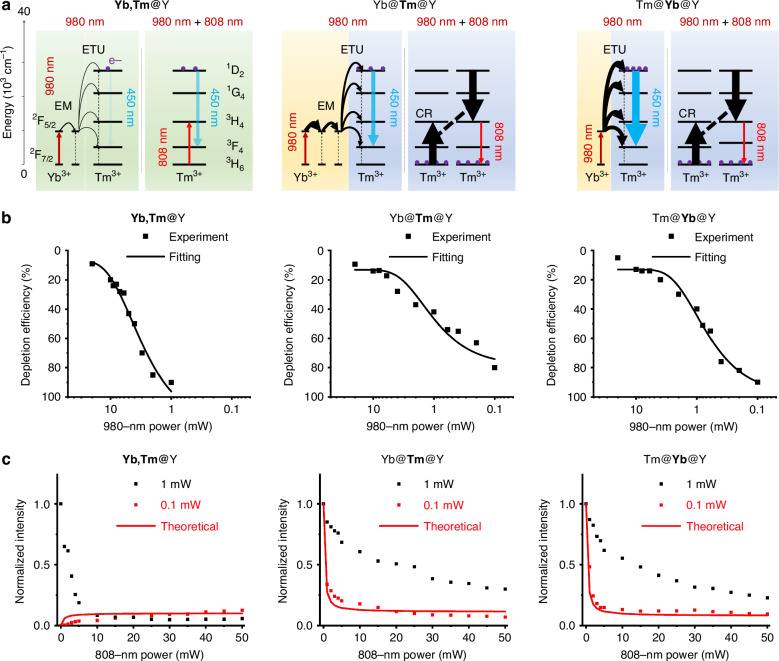
**Characterization of optical switching of UCL emission driven by topology-based ETNs in core-shell UCNPs under dual-beam irradiation for U-STED microscopy.** **a** Schematic energy level diagram of core-shell UCNPs illustrating the optical switching mechanism of 450-nm UCL emission mediated by topology-based ETNs under dual-beam irradiation. Key processes include EM within the Yb^3+^-Yb^3+^ network, ETU in the Yb^3+^-Tm^3+^ network, and CR in the Tm^3+^-Tm^3+^ network. The diagram compares two configurations: irradiation with a CW excitation laser at 980 nm (left) and dual-beam irradiation combining CW excitation and depletion lasers at 980 nm and 808 nm, respectively (right). **b** Depletion efficiency of 450-nm UCL emission from **Yb,Tm**@Y, Yb@**Tm**@Y, and Tm@**Yb**@Y UCNPs under dual-beam irradiation, using a CW excitation laser at 980 nm with varying power levels and a CW depletion laser at 808 nm at a fixed power of 10 mW. **c** Theoretical and experimental normalized intensity of 450-nm UCL emission from **Yb,Tm**@Y, Yb@**Tm**@Y, and Tm@**Yb**@Y UCNPs under dual-beam irradiation. Measurements were conducted with a CW excitation laser at 980 nm with power levels of 0.1 mW and 1 mW, combined with a CW depletion laser at 808 nm at varying power levels

Figure 3 illustrates the characterization of optical switching in UCL emission facilitated by topology-driven ETNs in core-shell UCNPs under dual-beam irradiation for U-STED microscopy. Figure 3a and S7 present schematic energy level diagrams of the core-shell UCNPs, depicting the optical switching mechanism of 450-nm UCL emission mediated by topology-driven ETNs under dual-beam irradiation. The key processes highlighted include EM within the Yb^3+^-Yb^3+^ network, ETU in the Yb^3+^-Tm^3+^ network, and CR pathways in the Tm^3+^-Tm^3+^ network. The diagrams compare two configurations: low-power irradiation with a CW excitation laser at 980 nm alone (left) and low-power dual-beam irradiation combining the CW excitation with depletion lasers at 980 nm and 808 nm, respectively (right). This comparison emphasizes the modulation of energy transfer pathways that control UCL emission and optical switching.

We conducted theoretical modeling to understand UCL emission in core-shell UCNPs driven by topology-based ETNs under dual-beam irradiation. A set of rate equations was developed to analyze the time-dependent electronic population distribution in these UCNPs, with dual-beam irradiation involving CW excitation and depletion laser at 980 nm and 808 nm, respectively (Table S3). The power levels considered for the excitation laser at 980 nm are 0.1 mW and 1 mW, while the depletion laser at 808 nm operates at 10 mW. Our theoretical model accounts for the distinct upconversion processes in typical, core-to-shell, and shell-to-core UCNP structures, despite having identical sensitizer-activator interfaces and concentrations. The differences in EM processes are critical, with shell-to-core UCNPs exhibiting fewer EM steps from Yb^3+^ sensitizers to Tm^3+^ emitters, compared to other UCNP configurations^34^. This difference results in reduced energy losses and a higher population of Tm^3+^ ions at the ^1^D₂ level in shell-to-core UCNPs, which in turn leads to stronger 450-nm UCL emission under low-power irradiation with a CW laser at 980 nm.

Extending this study to low-power dual-beam irradiation with both 980-nm and 808-nm CW lasers, we conducted theoretical modeling of the time-dependent electronic population distribution in core-shell UCNPs driven by topology-based ETNs under dual-beam irradiation. The system was excited with a 980-nm beam at powers of 0.1 mW and 1 mW, switched on at *t* = 0 ms, while an 808-nm depletion beam at a power of 10 mW was activated at *t* = 5 ms (Fig. S8). We observe an enhancement in the electronic population at the ^1^D₂ level of Tm^3+^ ions in **Yb,Tm**@Y UCNPs when the 808-nm CW laser is activated. This enhancement is attributed to upconversion, where Tm^3+^ ions directly absorb 808-nm photons, resulting in increased 450-nm UCL emission intensity. In contrast, in Yb@**Tm**@Y UCNPs, the activation of the CW laser at 808 nm induces depletion of the ^3^H_4_ level of Tm^3+^ ions and a reduction in the ^1^D₂ level population. This decrease is due to intense CR processes that cause population inversion in the intermediate excited state of Tm^3+^, enabling efficient optical switching of the 450-nm UCL emission. A similar behavior is observed in Tm@**Yb**@Y UCNPs, where intense CR processes lead to population inversion and efficient optical switching of the 450-nm UCL emission when the 808-nm laser is activated.

Under high-power irradiation with the 980-nm CW laser, all three UCNP structures exhibit population buildup at the ^1^D₂ level of Tm^3+^ ions, leading to intense 450-nm UCL emission. When both 980-nm and 808-nm CW lasers are used in high-power dual-beam irradiation, depletion of the ^3^H_4_ level of Tm^3+^ ions are observed, along with intense CR processes that induce population inversion in the intermediate excited state of Tm^3+^. This results in efficient optical switching of the 450-nm UCL emission, in line with previous reports^21,22^. Additionally, we observe an overlap between *n₁* (³H₆ ground state) and *n₃* (³H₄ intermediate state) of Tm^3+^ when the 808 nm depletion is activated, indicating a dynamic balance between absorption and STED^25^. This interplay further supports the accuracy and reliability of our theoretical model in describing the system’s behavior.

Based on these findings, we propose a framework for engineering the electronic population dynamics in core-shell UCNPs driven by topology-based ETNs. Our theoretical model suggests that the emitter@sensitizer@inert UCNP structure offers distinct advantages for U-STED microscopy, primarily due to enhanced 450-nm UCL emission under low-power 980-nm CW excitation and efficient optical switching of 450-nm UCL emission under both low-power 980-nm and 808-nm depletion lasers. This advantage arises from the topology-based shell-to-core ETN dynamics, which incorporate the following processes: 1) Yb^3+^-Yb^3+^ networks based on high Yb^3+^ doping in the intermediate shell facilitate absorption under low-power 980-nm irradiation and intense EM among Yb^3+^ sensitizers^41,42^; 2) Yb^3+^-Tm^3+^ networks across the core-shell interface enable efficient topology-driven ETU from Yb^3+^ sensitizers to Tm^3+^ emitters, leading to intense 450-nm UCL emission^39^; and 3) Tm^3+^-Tm^3+^ networks based on high Tm^3+^ doping in the core support robust CR processes among Tm^3+^ emitters, promoting population inversion and enabling efficient optical switching via STED under 808-nm depletion laser irradiation^21,22,25,26^.

Next, we proceed with the experimental characterization of optical switching of UCL emission driven by topology-based ETNs in core-shell UCNPs under dual-beam irradiation. Figure 3b quantifies the depletion efficiency of 450-nm UCL emission from **Yb,Tm**@Y, Yb@**Tm**@Y, and Tm@**Yb**@Y UCNPs under dual-beam irradiation, with the CW excitation laser at 980 nm operating at varying power levels and the 808-nm CW depletion laser fixed at 10 mW. We achieve a depletion efficiency of the UCL emission at 450 nm of 91% from **Yb,Tm**@Y UCNPs under dual-beam irradiation with the CW excitation laser at 980 nm operating at 1 mW, and 85% and 92% from Yb@**Tm**@Y and Tm@**Yb**@Y UCNPs under dual-beam irradiation with the CW excitation laser at 980 nm at 0.1 mW. Figure S9 illustrates the theoretical modeling of depletion efficiency in core-shell UCNPs, driven by topology-based ETNs under dual-beam irradiation. Figure 3c presents the theoretical and experimental normalized intensities of 450-nm UCL emission from **Yb,Tm**@Y, Yb@**Tm**@Y, and Tm@**Yb**@Y UCNPs under dual-beam irradiation. The measurements were performed using a CW excitation laser at 980 nm with power levels of 1 mW and 0.1 mW for **Yb,Tm**@Y, Yb@**Tm**@Y, and Tm@**Yb**@Y UCNPs, combined with a CW depletion laser at 808 nm operating at varying power levels. The 450-nm UCL emission intensity from **Yb,Tm**@Y UCNPs decreased when irradiated with a 980-nm CW excitation laser at 1 mW and an 808-nm CW depletion laser at varying powers, attributed to STED of these UCNPs, consistent with previous reports^25^. In contrast, the intensity increased under irradiation with a 980-nm CW excitation laser at 0.1 mW and an 808-nm CW depletion laser at varying powers, attributed to direct excitation of these UCNPs by the 808-nm CW laser, in line with previous studies^21,22^. The 450-nm UCL emission intensity from Yb@**Tm**@Y and Tm@**Yb**@Y UCNPs decreased under dual-beam irradiation, demonstrating that topology-driven ETNs in these UCNPs enable reduced excitation laser powers down to 0.1 mW and improved optical switching efficiency via STED under irradiation of low-power excitation and depletion lasers. We achieve a saturation intensity of 0.06 MW cm^−2^ under excitation at 980 nm and depletion at 808 nm for Tm@**Yb**@Y UCNPs. The reduced saturation intensity observed in emitter@sensitizer@inert UCNPs under low-power dual-beam irradiation indicates that the topology-driven ETN effect in core-shell UCNPs mitigates the constraints of the square root law followed by conventional super-resolution microscopy techniques. Both theoretical modeling and experimental results confirm a 92% depletion efficiency of UCL emission at 450 nm from Tm@**Yb**@Y UCNPs under dual-beam irradiation, achieved with a CW 980 nm excitation laser at 0.1 mW. These results highlight the effectiveness of core-shell UCNPs with topology-driven ETNs in achieving high depletion efficiencies under low-power dual-beam irradiation.

Modulation cycles of the 808-nm depletion laser, controlled by an optical shutter, enable precise “ON” and “OFF” switching of the 450-nm UCL emission from core-shell UCNPs under 980-nm excitation, as shown in Fig. S10. This demonstrates efficient and robust optical switching with precise dynamic control over multiple irradiation cycles. To further confirm this capability, we performed confocal microscopy imaging under CW excitation and depletion laser irradiation at 980 nm and 808 nm, respectively, detecting the 450-nm UCL emission from the core-shell UCNPs (Fig. S11). This confirms the effective optical switching of 450-nm UCL emission from core-shell UCNPs driven by topology-based ETNs under low-power dual-beam irradiation with both 980-nm CW and 808-nm CW lasers.

**Fig. 4:**
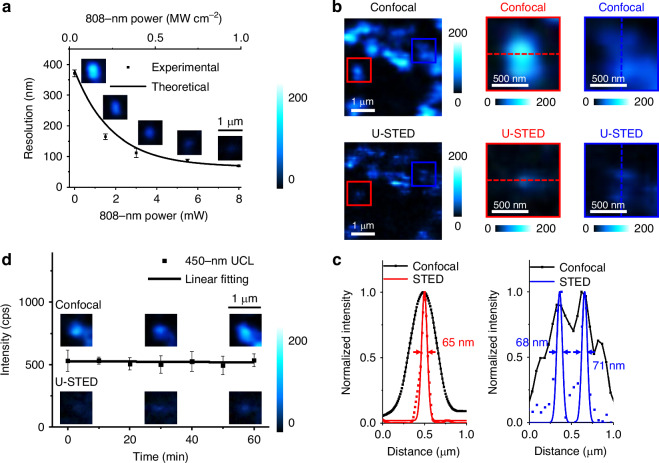
**U-STED microscopy of core-shell UCNPs with topology-driven ETNs.** **a** Comparison of the theoretical and experimental resolution under dual-beam irradiation. Resolution is measured using a CW Gaussian-shaped excitation laser at 980 nm with an intensity of 0.03 MW cm^−2^ and an CW donut-shaped depletion laser at 808 nm at increasing intensity levels. Insets: confocal and U-STED microscopy images of 450-nm UCL emission of core-shell Tm@**Yb**@Y UCNPs with topology-driven ETNs under dual-beam irradiation. **b** Confocal and U-STED microscopy images of 450-nm UCL emission from core-shell Tm@**Yb**@Y UCNPs with topology-driven ETNs under dual-beam irradiation using a CW Gaussian-shaped excitation laser at 980 nm and an CW donut-shaped depletion laser at 808 nm. Magnified views are shown (red and blue boxes). The measured intensities of the CW excitation and depletion lasers at 980 nm and 808 nm are 0.03 MW cm^−2^ and 1 MW cm^−2^, respectively. Pixel dwell time: 10 ms. Scale bar: 1 μm. **c** Normalized UCL emission intensity profiles corresponding to the dashed red and blue lines in (**b**). **d** Detection of 450-nm UCL emission from core-shell Tm@**Yb**@Y UCNPs with topology-driven ETNs under dual-beam irradiation for different duration. Inset: confocal and U-STED microscopy images of 450-nm UCL emission after 0 minutes, 30 minutes, and 60 minutes of dual-beam irradiation

Figure 4 highlights the application of U-STED microscopy for imaging core-shell UCNPs with topology-driven ETNs, specifically detecting the 450-nm UCL emission from Tm@**Yb**@Y UCNPs under dual-beam irradiation. The laser at 808 nm is spatially modulated to create a donut-shaped point spread function (PSF) that overlaps with the Gaussian PSF of the excitation laser at 980 nm at the focal plane (Fig. S12). This overlap selectively deactivates the UCNPs at the edges of the excitation laser, effectively shrinking the excitation spot size below the diffraction limit, consistent with STED microscopy principles.

Figure 4a compares the theoretical and experimental resolutions achieved under dual-beam irradiation. Measurements were conducted using a CW Gaussian-shaped excitation laser at 980 nm with an intensity of 0.03 MW cm^−2^ and an CW donut-shaped depletion laser at 808 nm with varying intensity levels. Insets provide representative confocal and U-STED microscopy images of the 450-nm UCL emission, illustrating significant resolution enhancement with increasing depletion laser intensity. Unlike conventional STED microscopy^12–14^, where excitation power is minimized to prevent nonlinear effects, U-STED systems require careful control of excitation intensity due to the nonlinear, multiphoton nature of the upconversion process^44^. Our theoretical simulations (Figs. S13 and S14), supported by experimental characterization, demonstrate that reducing the 980-nm excitation power results in a narrowing of the focal spot size^51^. This enhancement is primarily due to the suppression of nonlinear background emission and the avoidance of partial depletion saturation, both of which can broaden the effective PSF at higher excitation levels. These results highlight that operating below the excitation saturation threshold is essential for achieving optimal spatial resolution and contrast in U-STED imaging. Importantly, the simulations confirm that this low-excitation regime maintains sufficient upconversion efficiency for imaging, while significantly minimizing the resolution degradation associated with high excitation powers.

Figure 4b and S15 present comparisons of confocal and U-STED microscopy images of the 450-nm UCL emission. These images were captured using the same dual-laser setup, with measured intensities at the objective’s back aperture of 0.03 MW cm^−2^ for the excitation laser at 980 nm and 1 MW cm^−2^ for the depletion laser at 808 nm. Magnified views within the red and blue boxes emphasize the resolution improvements achieved with U-STED microscopy. Figure 4c shows normalized UCL emission intensity profiles corresponding to the dashed red and blue lines in Fig. 4b, quantitatively illustrating the spatial resolution improvement in U-STED imaging. Deconvolution and Gaussian fitting of the data reveal a full-width at half-maximum (FWHM) of 65 nm, confirming the superior resolution achieved. We imaged two UCNPs separated by approximately 200 nm. Using conventional confocal microscopy, we obtained a diffraction-limited image in which the two particles could not be resolved—only a single emission peak was visible, with a FWHM of around 780 nm in the corresponding cross-section. In contrast, U-STED microscopy produced a super-resolution image where the two UCNPs were clearly distinguishable, each displaying a distinct emission peak. The corresponding cross-sections revealed FWHMs of approximately 68 nm and 71 nm. These results highlight the capability of our U-STED approach to overcome the diffraction limit and deliver significantly enhanced spatial resolution.

A current limitation of our approach is the long emission lifetimes of the UCNPs used, typically on the order of hundreds of microseconds, which necessitate extended pixel dwell times and thus reduce imaging speed. This trade-off between low illumination intensity and temporal resolution constrains present U-STED microscopy. Recent progress in compositional engineering, such as high Yb^3+^ doping, has been shown to enhance emission intensity while shortening lifetimes, enabling dwell times as brief as 10 μs^25^. Additionally, advances in upconversion superfluorescence (SF) have achieved lifetimes below 50 ns^52^ and down to 2.5 ns^53^, opening new avenues for fast, low-power, high-resolution U-STED imaging.

Figure 4d evaluates the temporal stability of UCL emission under dual-beam irradiation at 0, 30, and 60-minute intervals. The results demonstrate the durability of topology-driven ETNs for prolonged imaging applications, with particle emission intensity decreasing at a gradual rate of only 2% per hour. Notably, the imaging resolution remains consistent throughout the duration, achieving a diffraction-limited resolution of less than 400 nm in the confocal regime and under 70 nm in the super-resolution U-STED regime. Particle alignment relative to the beams was verified at regular intervals to maintain stable imaging conditions.

## Discussion

Compared to conventional STED methods using molecular fluorophores, our U-STED approach presents distinct advantages. Recent developments in STED probes have enhanced imaging performance through greater brightness, photostability, spectral flexibility, and improved suitability for live-cell studies. Examples include monomeric NIR fluorescent proteins for multicolor imaging with reduced phototoxicity^54^, squaraine-based dyes for high-resolution mitochondrial visualization^55^, bright fluorophores with large Stokes shifts for improved nanoscopy^56^, and lipid-based probes enabling prolonged mitochondrial membrane imaging^57^. Despite these advances, traditional STED systems reliant on organic fluorophores still require high excitation and depletion power, which restricts their application in live or thick specimens due to increased photobleaching and phototoxicity.

The demonstrated reduction in excitation and depletion laser intensities is particularly impactful for biological imaging applications, where phototoxicity and photobleaching often limit live-cell and tissue studies. Our topology-driven ETNs in UCNPs enable super-resolution imaging with significantly reduced photodamage, making long-term and high-fidelity visualization of subcellular structures feasible. This advancement opens new opportunities for real-time imaging of dynamic biological processes at the nanoscale^58,59^, with potential applications ranging from neuroscience and cell biology to developmental and medical research^60–62^. Moreover, the NIR excitation wavelength used offers improved tissue penetration and minimal autofluorescence, further enhancing the suitability of our approach for in vivo imaging.

UCNPs offer several features beneficial for biological imaging^63,64^. Their surfaces can be engineered with antibodies, peptides, or oligonucleotides to enable selective targeting of proteins, nucleic acids, or organelles with high specificity^65^. Surface charge and hydrophilicity modulate cellular uptake pathways, allowing optimization for different cell types^66^. Although larger than small-molecule dyes, UCNPs can be synthesized with hydrodynamic diameters below 50 nm, facilitating endocytosis and minimizing steric interference. Their intrinsic NIR-excited luminescence enables label-free imaging, reducing autofluorescence and phototoxicity. Importantly, studies have demonstrated minimal cytotoxicity and negligible perturbation of cellular processes when UCNPs are properly coated and dosed^67–69^. Despite challenges in achieving efficient intracellular delivery and minimizing off-target effects, these attributes support the potential of UCNPs as viable probes for super-resolution imaging of biological structures.

Beyond life sciences, the ability to achieve efficient upconversion and optical switching at low laser powers through topology-engineered ETNs paves the way for integration of UCNPs into nanophotonic devices. These include energy-efficient nanoscale light sources^70^, optical modulators^71^, and switches^72,73^ essential for next-generation optical communication and computing technologies^74,75^. Moving forward, the employment of smaller and brighter lanthanide-doped UCNPs with robust optical stability is critical for expanding the applications of low-power U-STED microscopy based on our approach. Recent advances have introduced strategies to synthesize ultrasmall UCNPs (less than 10 nm in size) with enhanced brightness and stability. These methods include optimizing the host matrix, tailoring lanthanide ion doping concentrations, applying inert shell coatings, and precisely controlling surface interactions^76–78^.

Studying the energy transfer dynamics and directions within UCNPs, along with the spatial distribution, combinations, and concentration of lanthanide ions, is crucial for advancing super-resolution applications that rely on low laser intensities. These results provide deeper insights into these energy transfer mechanisms^79,80^ and underscore the significance of controlled synthesis methods^81,82^, especially for heterogeneous lanthanide-doped UCNPs^83–86^. Our findings open new avenues for advanced super-resolution techniques with multi-modality, leveraging lanthanide-doped UCNPs to enhance their versatility in state-of-the-art optical imaging systems^87,88^. The ability to engineer nanocrystals with tailored optical properties will pave the way for next-generation UCNPs, enhancing the efficiency of super-resolution imaging and characterization^89–91^. These advancements in topology-dependent energy transfer within UCNPs could significantly reduce laser intensity requirements, thereby expanding the potential of UCNPs for low-power super-resolution applications in life science^92–94^ and nanophotonics^95–100^.

## Materials and methods

### Sample preparation

Lanthanide-doped core-shell UCNPs are synthesized following previously established protocols with minor modifications^101^. These UCNPs, obtained from Xi’an Ruixi Biological Technology Co., Ltd. (http://www.xarxbio.com), are used as received without additional processing. The nanoparticle compositions are as follows: 1) NaYF₄:25%Yb,5%Tm@NaYF₄, 2) NaYF₄:25%Yb@5%Tm@NaYF₄, and 3) NaYF₄:5%Tm@25%Yb@NaYF₄. Core-shell UCNPs with thin core and intermediate shells are referred to as Yb,Tm@Y, Yb@Tm@Y, and Tm@Yb@Y UCNPs, while those with thick core and intermediate shells are labeled as **Yb,Tm**@Y, Yb@**Tm**@Y, and Tm@**Yb**@Y UCNPs.

Sample slides containing individually distributed UCNPs are carefully prepared using a standardized procedure. A coverslip is first cleaned by ultrasonication in pure ethanol and Milli-Q water, then air-dried. A 20 μL droplet of UCNPs, diluted to a concentration of 0.01 mg/mL in cyclohexane, is applied to the surface of the coverslip. The sample is immediately rinsed twice with 500 μL of cyclohexane to ensure uniform distribution. Finally, the prepared slides are left to dry at room temperature for 24 hours before measurement.

### Sample characterization

Transmission electron microscopy (TEM) was conducted using a JEOL JEM 2100 F, operating at an accelerating voltage of 200 kV and equipped with an 11-megapixel bottom-mounted Quemesa camera. High-resolution TEM imaging is conducted with a JEM-F200(URP) TEM, also at an acceleration voltage of 200 kV. X-ray powder diffraction (XRD) analysis is carried out using a Rigaku Ultima IV diffractometer with a Cu Kα radiation source (λ = 1.54056 Å). Upconversion luminescence (UCL) emission spectra are acquired using an Andor Shamrock 500i imaging spectrometer, coupled with a Leica DFC295 digital color camera, and excited by a 980-nm continuous-wave (CW) laser (Thorlabs, BL976-PAG900).

### Dual-beam super-resolution optical system setup

The dual-beam super-resolution optical system setup for U-STED microscopy is developed based on a custom-built confocal microscopy system. Samples are mounted on a computer-controlled, high-precision nanopositioning system utilizing a three-axis piezostage (Physik Instrumente, P-562.3CD). A CW laser at 980 nm (Micro Photons Technology Co., COBRITE-980-M) serves as the excitation source for the UCNPs. After collimation, the 980-nm CW laser passes through two long-pass dichroic mirrors and is focused onto the sample using an oil-immersion objective lens (Olympus, UPlanApo 100X/1.5 oil). The first dichroic mirror (DC1, Semrock, FF880-SDi01-t3-25×36) combines the CW laser at 980 nm with a collimated CW laser at 808 nm (Obis, OBIS-LX-808) for depletion.

The UCL emission from the UCNPs is collected by the same objective lens and separated from the 980-nm CW excitation and 808-nm CW depletion lasers using a second dichroic mirror (DC2, Semrock, Di03-R785-t3-25 × 36). The emission is then coupled into a multimode fiber (Thorlabs, FG050LGA) connected to a single-photon avalanche diode (SPAD, Laser Components, Count T100-FC). To isolate the desired UCL emission bands for imaging, a bandpass filter (Semrock, FF01-448/20-25) and a short-pass filter (Semrock, BSP01-785R-25) are placed in the detection pathway. For super-resolution imaging, a quarter-wave plate (Thorlabs, WPQ10M-808) converts the 808-nm CW laser into circularly polarized light. A half-wave plate (Thorlabs, WPH10M-850) is used in conjunction with Glan-Thompson prisms (Thorlabs, DGL10) to control laser power. Finally, a vortex phase plate (UPOLabs, LETO-808-C) is incorporated into the 808-nm CW laser path to generate a donut-shaped PSF in the focal plane.

### UCL emission lifetime measurement system setup

For the measurement of UCL emission lifetime, a 980-nm CW laser (Thorlabs, BL976-PAG900) is employed. This laser is integrated into a commercial optical microscopy system setup (Leica DM2700M microscope) equipped with time-correlated single-photon counting (TCSPC) equipment (Zolix, DCS900PC). The 980-nm CW laser is modulated using a waveform generator (RIGOL, DG5072) to produce 10-μs pulses at a frequency of 500 Hz, facilitating the excitation of UCL emission. Emitted photons are filtered through a bandpass filter (Semrock, SP01-785RU-25) and a short-pass filter (Semrock, FF01-448/20-25) before being detected by the TCSPC system. The effective UCL emission decay time (*τ*_*eff*_) is then calculated using the collected data as follows:

1 $${\tau }_{{eff}}=\frac{1}{{I}_{0}}{\int }_{0}^{\infty }I\left(t\right){dt}$$

where *I(t)* is the emission intensity as a function of time *t* and *I*_*0*_ are the maximum emission intensity.

### Calculation of lanthanide ion quantity in core-shell UCNPs

Accurately determining the quantity of lanthanide ions in core-shell UCNPs is essential for analyzing their UCL emission, particularly for applications in U-STED microscopy. We calculate the number of Yb^3+^ and Tm^3+^ ions in core-shell UCNPs, considering particle geometry, crystal structure, and doping concentrations. The calculations are illustrated for three distinct core-shell UCNP configurations. We assume the UCNPs are spherical, with the volume *V*_*ucnp*_ calculated as:

2 $${V}_{{ucnp}}=\frac{4\pi {r}^{3}}{3}$$

where *r* is the radius of the UCNP. For core-shell structures, the core volume *V*_*core*_ and shell volume *V*_*shell*_ are calculated separately as:

3 $${V}_{{core}}=\frac{4\pi {r}_{{core}}^{3}}{3}$$

4 $${V}_{{shell}}={V}_{{ucnp}}-{V}_{{core}}$$

For UCNP with *β*-phase consisting of hexagonal unit cells, the volume of a hexagonal unit cell *μV*_*hexagonal*_ is given by:

5 $${\rm{\mu }}{V}_{{hexagonal}}=\frac{2\sqrt{3}{a}^{3}c}{2}$$

Where *a* = 5.91 Å and *c* = 3.53 Å are lattice parameters describing hexagonal unit cells. Then, the number of unit cells in a UCNP is estimated by:

6 $${\rm{\mu }}{N}_{{hexagonal}}=\frac{{V}_{{region}}}{{\rm{\mu }}{V}_{{hexagonal}}}$$

where is the volume of the core, shell, or total UCNP. Each hexagonal unit cell contains four formula units of NaYF₄. The number of formula units in a given region is:

7 $${N}_{{formula}\_{units}}=4\times {\rm{\mu }}{N}_{{hexagonal}}$$

Yb^3+^ ions and Tm^3+^ ions replace Y^3+^ ions in NaYF₄ formula units according to the doping percentage. For a doping ratio $x$%, the number of ions is:

8 $${N}_{{ions}}={N}_{{formula}\_{units}}\times x \%$$

The calculated ion quantities for three different UCNP configurations are reported in Table S2.

### Theoretical modeling of UCL emission in core-shell UCNPs driven by topology-based energy transfer networks (ETNs) under dual-beam irradiation

The theoretical modeling of UCL emission in core-shell UCNPs driven by topology-based ETNs under dual-beam irradiation is conducted by developing a set of rate equations. These equations account for the topology-dependent energy migration (EM) within Yb^3+^-Yb^3+^ ETNs, energy transfer upconversion (ETU) within Yb^3+^-Tm^3+^ ETNs, and cross-relaxation (CR) processes within Tm³⁺-Tm³⁺ ETNs. This approach assumes rapid non-radiative decay for the transitions from ^1^H_5_ to ^3^F_4_ and from ^3^F_2,3_ to ^3^H_4_ in Tm^3+^ ions, effectively merging each pair of energy levels into a single level for simplification.

For Yb^3+^-Yb^3+^ ETNs:

• Yb^3+^ (^2^F_7/2_), ground-state *n*_*Y1*_:

9 $$\frac{d{n}_{Y1}}{{dt}}=-\frac{d{n}_{Y2}}{{dt}}$$

• Yb^3+^ (^2^F_5/2_), excited-state *n*_*Y2*_:

10 $$\frac{d{n}_{Y2}}{{dt}}={P}_{980}{n}_{Y1}-{W}_{s}{n}_{Y2}-{EM}\times {n}_{Y2}$$

For Yb^3+^-Tm^3+^ and Tm-Tm^3+^ ETNs:

• Yb^3+^ (^2^F_7/2_), ground-state *n*_*S1*_:

11 $$\frac{d{n}_{S1}}{{dt}}=-\frac{d{n}_{S2}}{{dt}}$$

• Yb^3+^ (^2^F_5/2_), excited-state *n*_*S2*_:

12 $$\frac{d{n}_{S2}}{{dt}}={P}_{980}{n}_{S1}-{W}_{s}{n}_{S2}+{EM}\times {n}_{Y2}-\left({c}_{1}{n}_{1}+{c}_{2}{n}_{2}+{c}_{3}{n}_{3}+{c}_{4}{n}_{4}\right){n}_{S2}$$

• Tm^3+^ (^3^H_6_), ground-state *n*_*1*_:

13 $$\frac{d{n}_{1}}{{dt}}=-\mathop{\sum }\limits_{i=2}^{5}\frac{d{n}_{i}}{{dt}}$$

• Tm^3+^ (^3^F_4_, ^3^H_5_), excited-state *n*_*2*_:

14 $$\frac{d{n}_{2}}{{dt}}={c}_{1}{n}_{S2}{n}_{1}-{c}_{2}{n}_{S2}{n}_{2}-{W}_{2}{n}_{2}+{b}_{32}{W}_{3}{n}_{3}+{b}_{42}{W}_{4}{n}_{4}+{b}_{52}{W}_{5}{n}_{5}+2{k}_{31}{n}_{1}{n}_{3}+{k}_{41}{n}_{1}{n}_{4}$$

• Tm^3+^ (^3^H_4_, ^3^F_2,3_), excited-state *n*_*3*_:

15 $$\frac{d{n}_{3}}{{dt}}={{P}_{808}\left({n}_{1}-{n}_{3}\right)+c}_{2}{n}_{S2}{n}_{2}-{c}_{3}{n}_{S2}{n}_{3}-{W}_{3}{n}_{3}+{b}_{43}{W}_{4}{n}_{4}+{b}_{53}{W}_{5}{n}_{5}-{k}_{31}{n}_{1}{n}_{3}+{k}_{41}{n}_{1}{n}_{4}+2{k}_{51}{n}_{1}{n}_{5}$$

• Tm^3+^ (^1^G_4_), excited-state *n*_*4*_:

16 $$\frac{d{n}_{4}}{{dt}}={c}_{3}{n}_{S2}{n}_{3}-{c}_{4}{n}_{S2}{n}_{4}-{W}_{4}{n}_{4}+{b}_{54}{W}_{5}{n}_{5}-{k}_{41}{n}_{1}{n}_{4}$$

• Tm^3+^ (^1^D_2_), excited-state *n*_*5*_:

17 $$\frac{d{n}_{5}}{{dt}}={c}_{4}{n}_{S2}{n}_{4}-{W}_{5}{n}_{5}-{k}_{51}{n}_{1}{n}_{5}$$

where ${P}_{980}=\left(\frac{{\sigma }_{{Yb}}{\lambda }_{980}{I}_{980}}{{hc}}\right)$ is the excitation rate of Yb^3+^ ions under irradiation at the wavelength of 980 nm, and *I*_*980*_ is the intensity of the laser at the wavelength of 980 nm for excitation; ${P}_{808}=\left(\frac{{\sigma }_{{STED}}{\lambda }_{808}{I}_{808}}{{hc}}\right)$ is the absorption/stimulated emission rate of Tm^3+^ ions under irradiation at the wavelength of 808 nm, so that the term of $\left(\frac{{\sigma }_{{STED}}{\lambda }_{808}{I}_{808}}{{hc}}\right)\left({n}_{1}-{n}_{3}\right)$ is the net effect of absorption/stimulated emission, and *I*_808_ is the intensity of the laser at the wavelength of 808 nm for depletion; *σ*_*Yb*_ is the absorption cross-section of Yb^3+^ ions for a laser at 980 nm; *σ*_*STED*_ is the absorption/stimulated emission cross-section of Tm^3+^ ions for a laser at 808 nm; *λ*_*980*_ is the wavelength of the laser for excitation; *λ*_*808*_ is the wavelength of the laser for depletion; *h* is the Planck’s constant; *c* is the light speed; *W*_S_ is the intrinsic decay rate of the excited Yb^3+^ ions; *c*_*i*_ is the upconversion coefficient between the excited Yb^3+^ ions and Tm^3+^ ions on level *i*; *W*_*i*_ is the intrinsic decay rate of Tm^3+^ ions on level *i*; *b*_*ij*_ is the branching ratio for Tm^3+^ ions decaying from level *i* to level *j*, satisfying ${\sum }_{j=1}^{i-1}{b}_{{ij}}=1$; *k*_*ij*_ is the CR coefficient between Tm^3+^ ions on level *i* and level *j*; *EM* is the EM coefficient between Yb^3+^ ions; and *n* is the electronic population in a specific energy level fulfilling the following conditions:

18 $${n}_{Y1}+{n}_{Y2}=1$$

19 $${n}_{S1}+{n}_{S2}=1$$

20 $${n}_{1}+{n}_{2}+{n}_{3}+{n}_{4}+{n}_{5}=1$$

The theoretical model presented here involves parameters specific to the properties of core-shell UCNPs. The reaction constants are fixed, leaving only *I*_*980*_ and *I*_*808*_ as variables. To validate the theoretical model, the UCL emission driven by topology-based ETNs of core-shell UCNPs is simulated using the parameters in Table S3.

### Characterizing UCL emission via power-law dependence

The intensity of UCL emission, *I*_*UCL*_, generally follows a power-law dependence on the excitation power density, *P*, expressed as *I*_*UCL*_ ∝ *Pⁿ*, where the exponent *n* indicates the number of photons participating in the multiphoton absorption process^44^. To determine the exponent *n*, *I*_*UCL*_ was measured at various levels of *P*. By applying a logarithmic transformation, this equation can be expressed in linear form as log (*I*_*UCL*_) = *n* log (*P*) + constant. A plot of log (*I*_*UCL*_) versus log (*P*) yields a straight line, where the slope of the fitted line corresponds to the exponent *n*. This value is determined through linear regression of the log-transformed data. In an ideal multiphoton excitation process, *n* is expected to be an integer, indicating the number of photons involved in excitation. However, deviations from integer values can occur due to factors such as saturation effects, energy transfer, or non-radiative relaxation mechanisms. By analyzing *n*, the excitation dynamics and energy transfer processes underlying UCL emission were characterized.

### Simulated resolution for U-STED microscopy using core-shell UCNPs driven by topology-based ETNs

STED microscopy is a super-resolution imaging technique that combines UCL emission with STED principles to achieve spatial resolution beyond the light diffraction limit. Like conventional STED microscopy, it utilizes a dual-beam optical system consisting of a Gaussian-shaped excitation laser and a donut-shaped depletion laser. These lasers are spatially overlapped to confine the PSF to the nanoscale center of the donut, enabling precise super-resolution imaging. The established theoretical model of UCL emission driven by topology-based ETNs is applied to calculate the electron populations in the energy levels of core-shell UCNPs. By simulating the intensity distribution of the Gaussian-shaped excitation laser at 980 nm and the donut-shaped depletion laser at 808 nm in the focal region, the achievable resolution under dual-beam super-resolution irradiation is evaluated. This setup uses a circularly polarized excitation laser at 980 nm and a circularly polarized vortex depletion laser at 808 nm. The vortex beam is generated with a 2π vortex phase plate, which imposes a helical phase retardation from 0 to 2π, and is focused using a high numerical aperture (NA) objective lens. The resulting electric field distribution in the focal region is expressed as^102^:

21 $$E\left(r,\varphi ,z\right)=\left[\begin{array}{c}{E}_{x}\\ {E}_{y}\\ {E}_{z}\end{array}\right]=-\frac{{ikf}}{2\pi }{\int }_{0}^{\alpha }{\int }_{0}^{2\pi }A\left(\theta \right)\exp \left({im}\phi \right)\sin \theta \sqrt{\cos \theta }\times \exp \left[{ik}\left(z\cos \theta +r\sin \theta \cos \left(\phi -\varphi \right)\right)\right]\times \left[\begin{array}{c}\left({\cos }^{2}\phi \cos \theta +{\sin }^{2}\phi \right)\pm i\cos \phi \sin \phi \left(\cos \theta -1\right)\\ \cos \phi \sin \phi \left(\cos \theta -1\right)\pm i\left({\cos }^{2}\phi +{\sin }^{2}\phi \cos \theta \right)\\ \sin \theta \exp \left(\pm i\phi \right)\end{array}\right]d\theta d\phi$$

Here, *r*, and *z* are the cylindrical coordinates, is the azimuthal angle of the incident laser, is the wave vector, *f* is the focal length of the high NA objective, *α* is the maximal NA angle, and *θ* is the NA angle that varies between 0 and *α*. Assuming that the optical system is in free space (i.e., the index of refraction *n* is 1), the maximal angle *α* is given by *α* = arcsin (*NA*/*n*), where *n* is the index of refraction of the material in the focal region. *A(θ)* is the pupil apodization function at the surface of the objective’s aperture, and exp(*im*$\phi$) is the vortex phase factor of the incident laser. For the excitation laser, *m* = 0, while for the depletion laser passing through the 2π phase plate, *m* = 1. The symbol relates to the handedness of the circularly polarized lasers. Right circularly polarized lasers are considered in this calculation. For the focal spot calculation, the considered NA for the objective lens is 1.5.

## Supplementary information

Supporting Information

## Data Availability

The data that support the findings of this study are available from the corresponding author upon reasonable request.
