# Supplementary material for: Topology-driven energy transfer networks for upconversion stimulated emission depletion microscopy
Source: Light Sci Appl. 2025 Dec 4;14:395. doi: 10.1038/s41377-025-02054-y (PMC12675517; doi:10.1038/s41377-025-02054-y)
Supplement: Supplementary file 1 — Supporting Information [file 41377_2025_2054_MOESM1_ESM.docx]

**Supplementary Information for**

Topology-driven energy transfer networks for upconversion stimulated emission depletion microscopy

Weizhao Gu#, Simone Lamon#*, Haoyi Yu, Qiming Zhang*, Min Gu*

#These authors contributed equally: Weizhao Gu and Simone Lamon.

W. Gu, S. Lamon, H. Yu, Q. Zhang, M. Gu

School of Artificial Intelligence Science and Technology, University of Shanghai for Science and Technology, Shanghai, 200093 China.

Institute of Photonic Chips, University of Shanghai for Science and Technology, Shanghai, 200093 China.

E-mail: simonelamon@usst.edu.cn, qimingzhang@usst.edu.cn, gumin@usst.edu.cn

**
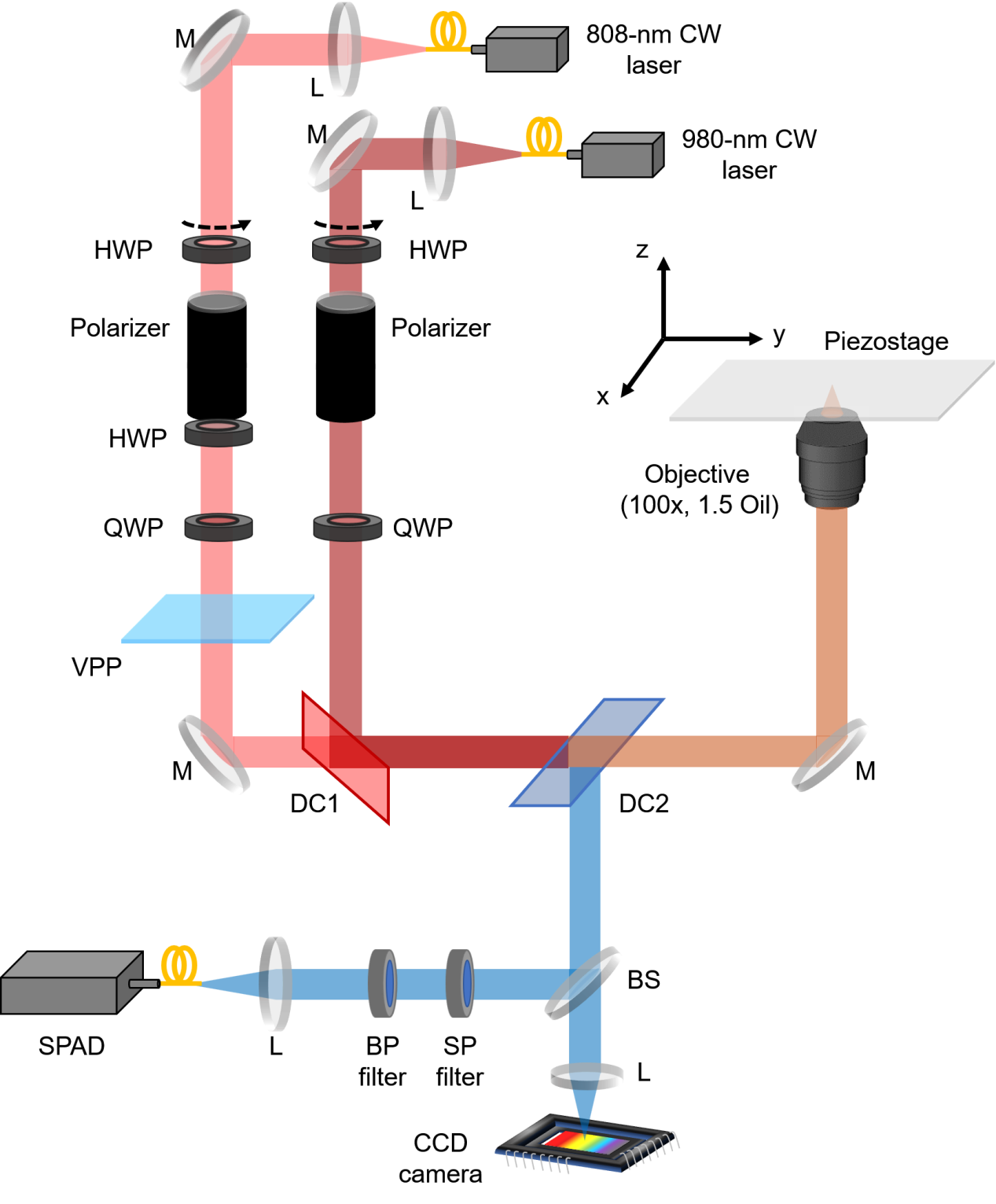
**

**Figure S1:** Schematic of the experimental dual-beam super-resolution optical system setup: M = mirror, L = lens, DC1 and DC2 = dichroic mirrors, BP filter = band pass filter, SP filter = short pass filter, HWP = half-wave plate, QWP = quarter-wave plate, VPP = vortex phase plate, BS = beam splitter, SPAD = single-photon avalanche diode, CCD camera = charge-coupled device camera.


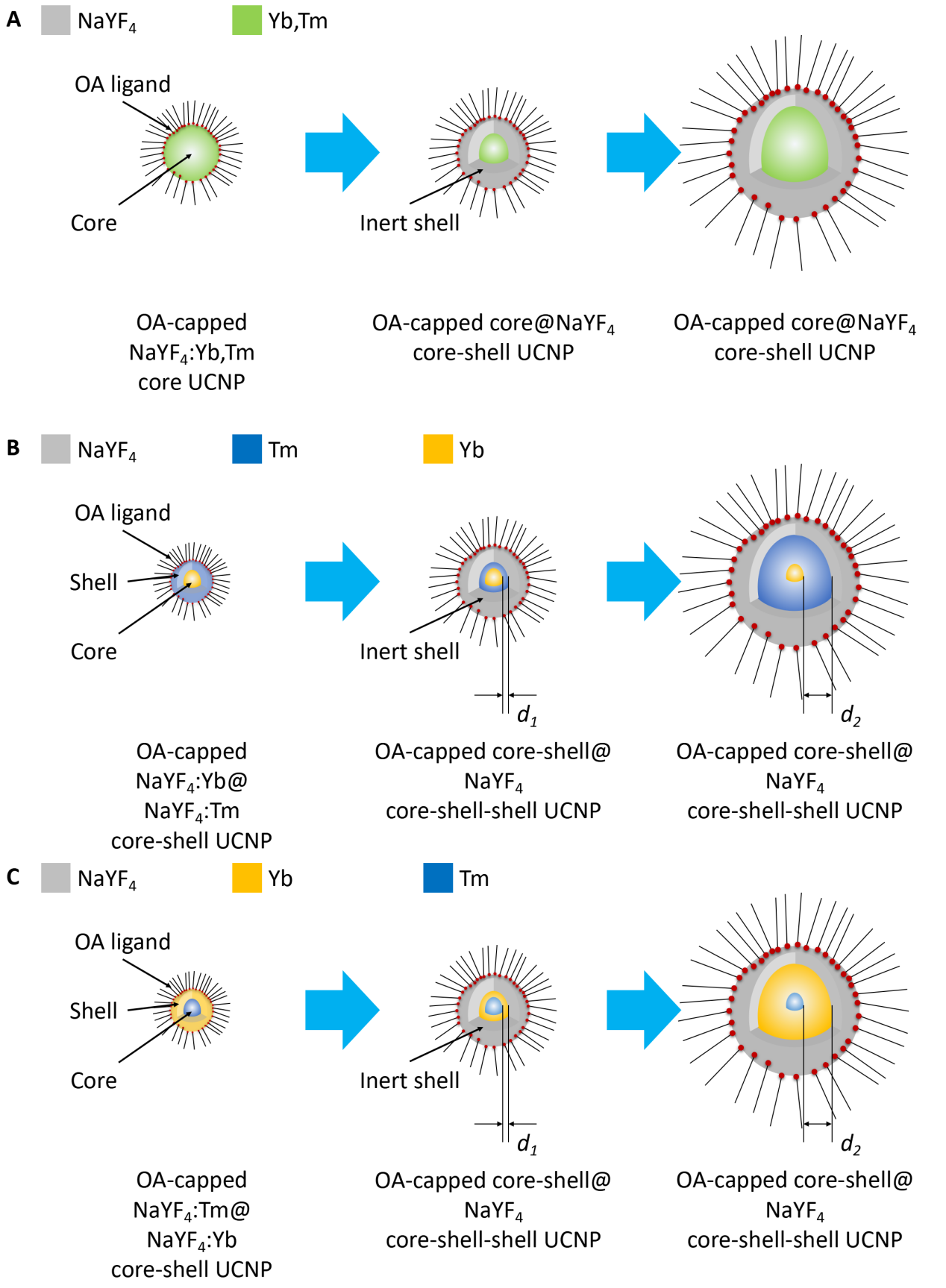


**Figure S2:** Schematic of the synthesis of the core-shell UCNPs. **A**) Schematic of the synthesis of NaYF_4_:Yb,Tm@NaYF_4_ UCNPs with thin and thick cores. **B**) Schematic of the synthesis of NaYF_4_:Yb@NaYF_4_:Tm@NaYF_4_ UCNPs with thin and thick intermediate shells. **C**) Schematic of the synthesis of NaYF_4_:Tm@NaYF_4_:Yb@NaYF_4_ UCNPs with thin and thick intermediate shells. OA = oleic acid. *d* = intermediate shell thickness.


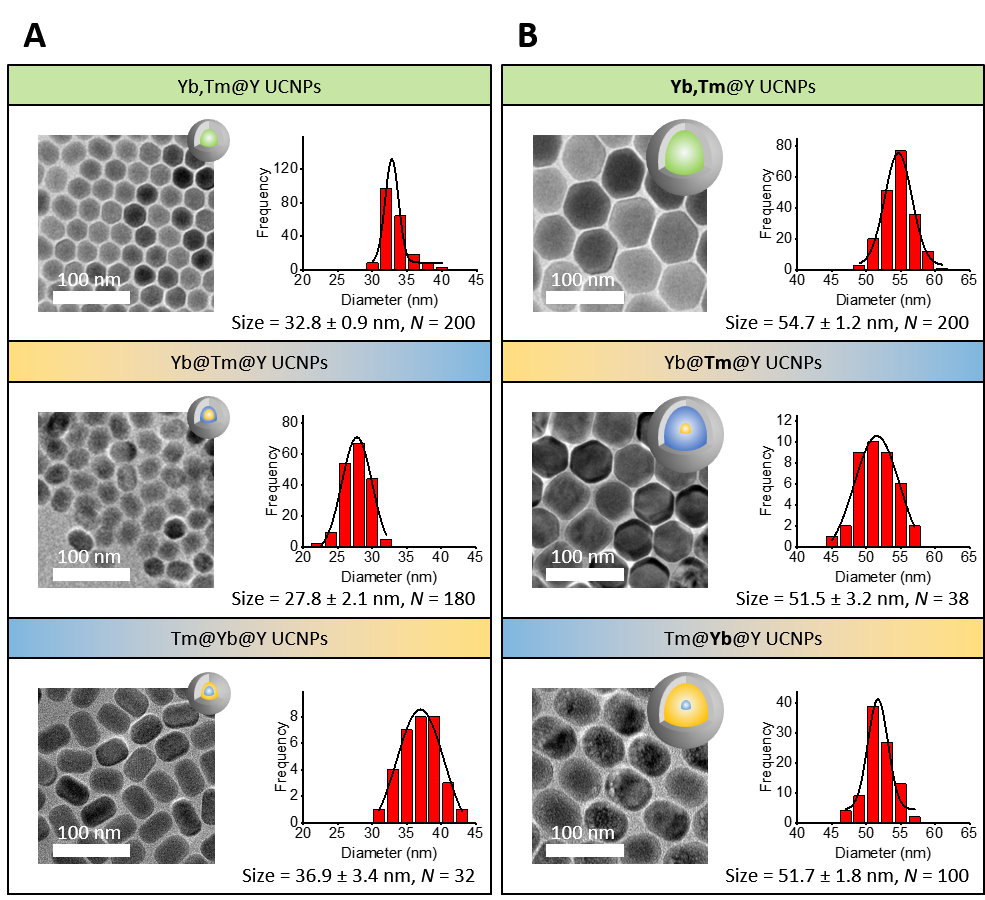


**Figure S3:** TEM imaging and size distribution of the core-shell UCNPs. **A**) TEM imaging and size distribution of the core-shell UCNPs with thin core and intermediate shells (from top to bottom, Yb,Tm@Y, Yb@Tm@Y, and Tm@Yb@Y UCNPs). **B**) TEM imaging and size distribution of the core-shell UCNPs with thick core and intermediate shells (from top to bottom, **Yb,Tm**@Y, Yb@**Tm**@Y, and Tm@**Yb**@Y UCNPs). *N* indicates the number of core-shell UCNPs considered for the calculation of the size distribution. Scale bar: 100 nm.


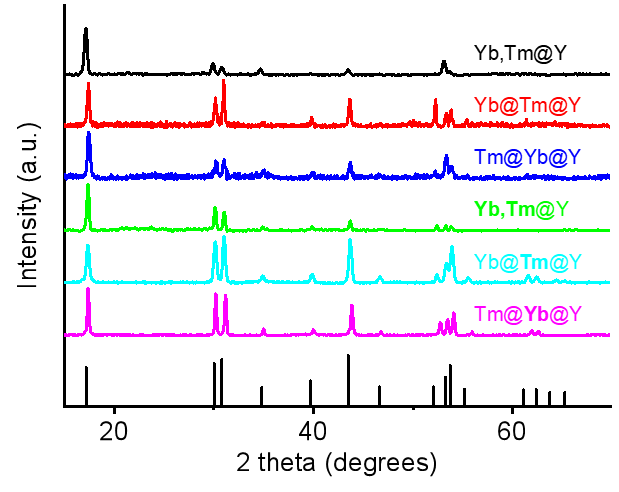


**Figure S4:** XRD spectra of the core-shell UCNPs (from black to purple solid lines, Yb,Tm@Y, Yb@Tm@Y, Tm@Yb@Y, **Yb,Tm**@Y, Yb@**Tm**@Y, and Tm@**Yb**@Y UCNPs) and corresponding standard pattern of hexagonal phase of β-NaYF_4_ (ICDD PDF 16-334).

**
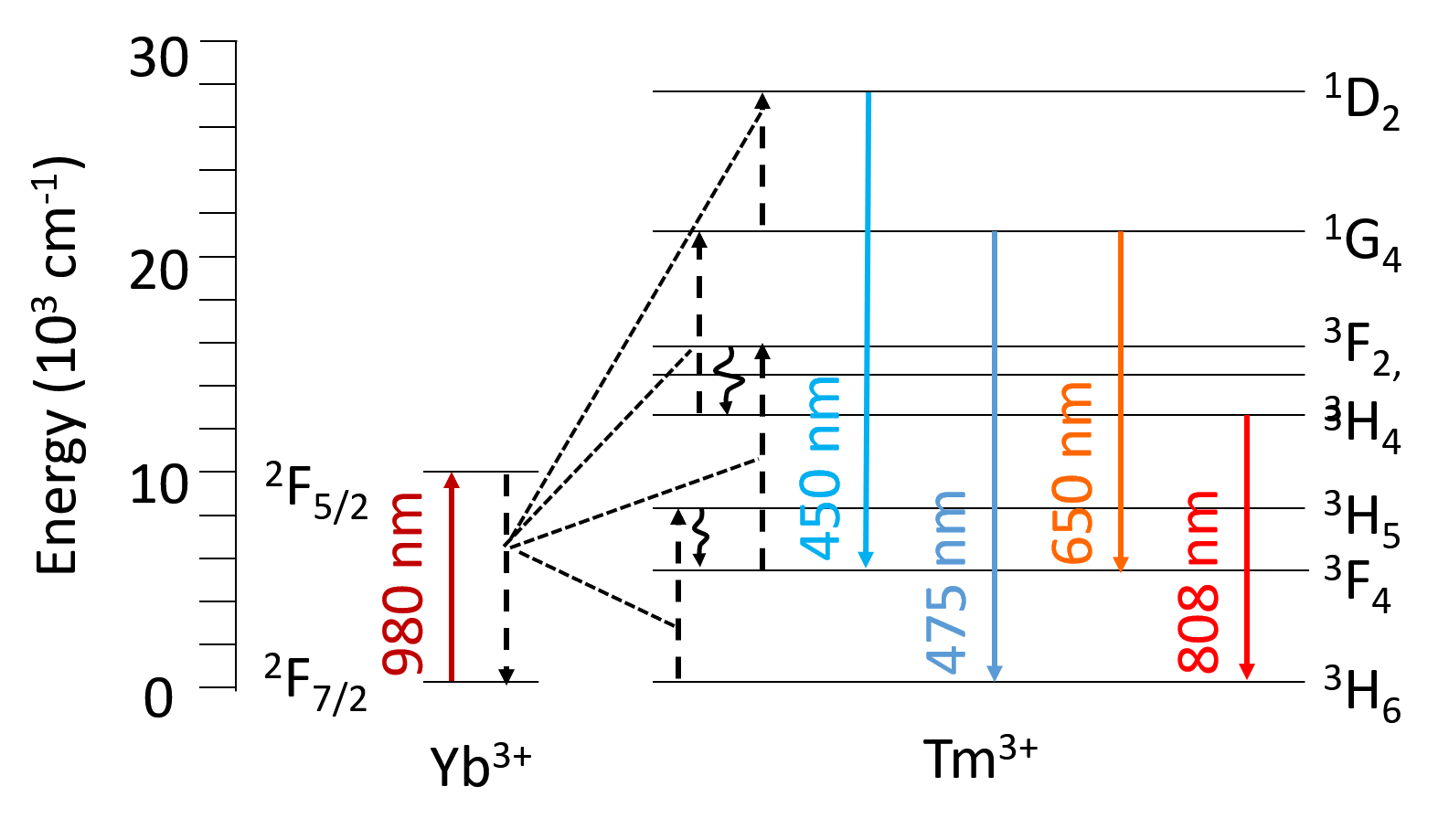
Figure S5:** Schematic of the energy level diagram of Yb³⁺ and Tm³⁺ ions in core-shell UCNPs and the proposed mechanism for UCL emission under irradiation with a 980-nm CW excitation laser. The Yb³⁺ ions absorb excitation photons at 980 nm and transfer energy quanta to the Tm³⁺ ions, facilitating upconversion from the ^3^H_6_ ground state to the ^1^D_2_ excited state via ETU. UCL emission at 450 nm originates from the transition between ^1^D_2_ and ^3^F_4_, while emissions at 475 nm, 650 nm, and 808 nm result from the transitions between ^1^G_4_ and ^3^H_6_, ^1^G_4_ and ^3^F_4_, and ^3^H_4_ and ^3^H_6_ of Tm³⁺ ions, respectively.


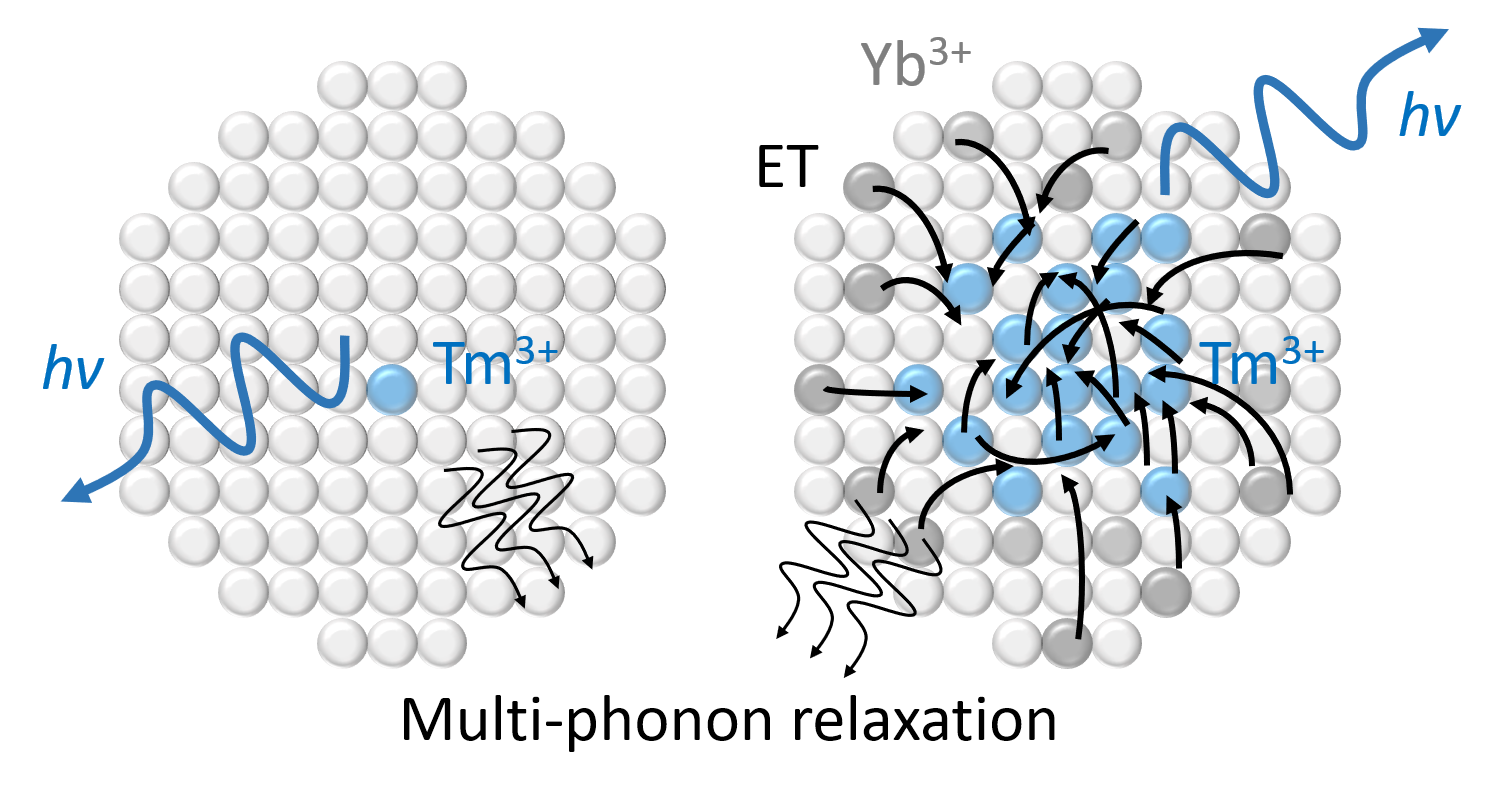


**Figure S6:** Schematic of an isolated Tm³⁺ ion within a UCNP (representing a single emitter), showing its possible relaxation pathways (left), and a schematic of a network of Yb³⁺-Tm³⁺ and Tm³⁺-Tm³⁺ ions within a UCNP (right). ET = energy transfer.


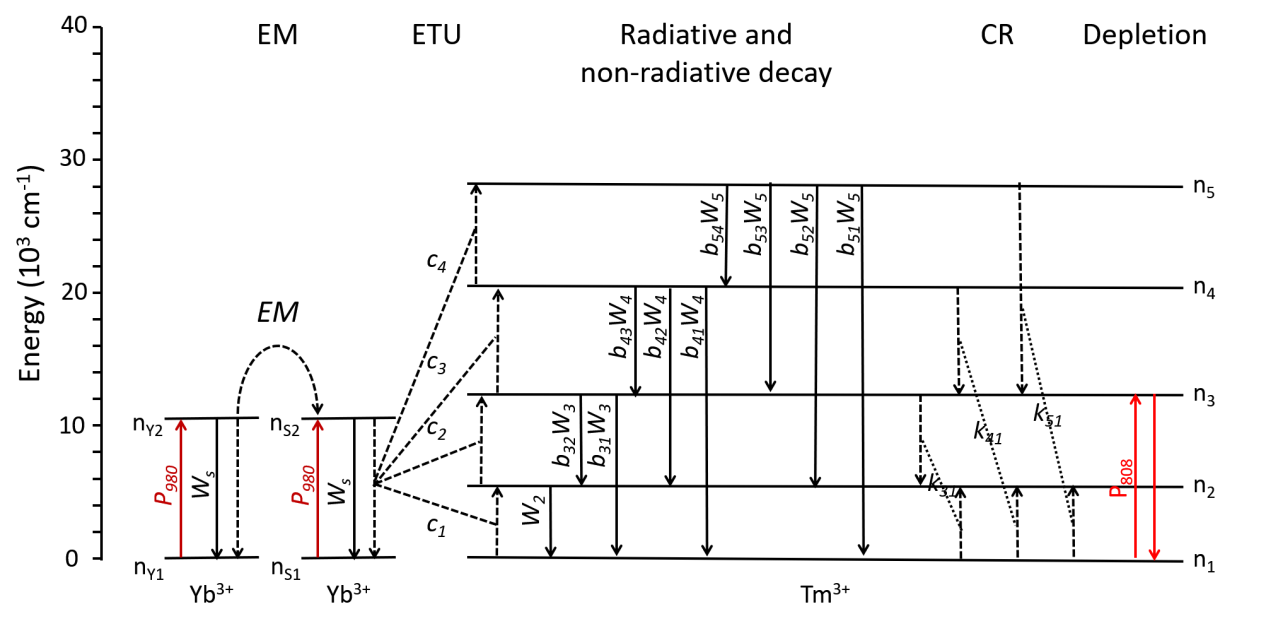


**Figure S7:** Schematic of the energy level diagram for the theoretical modeling of UCL emission in core-shell UCNPs driven by topology-based ETNs under dual-beam irradiation, consisting of excitation at 980 nm and depletion at 808 nm. EM = energy migration, ETU = energy transfer upconversion, CR = cross-relaxation.


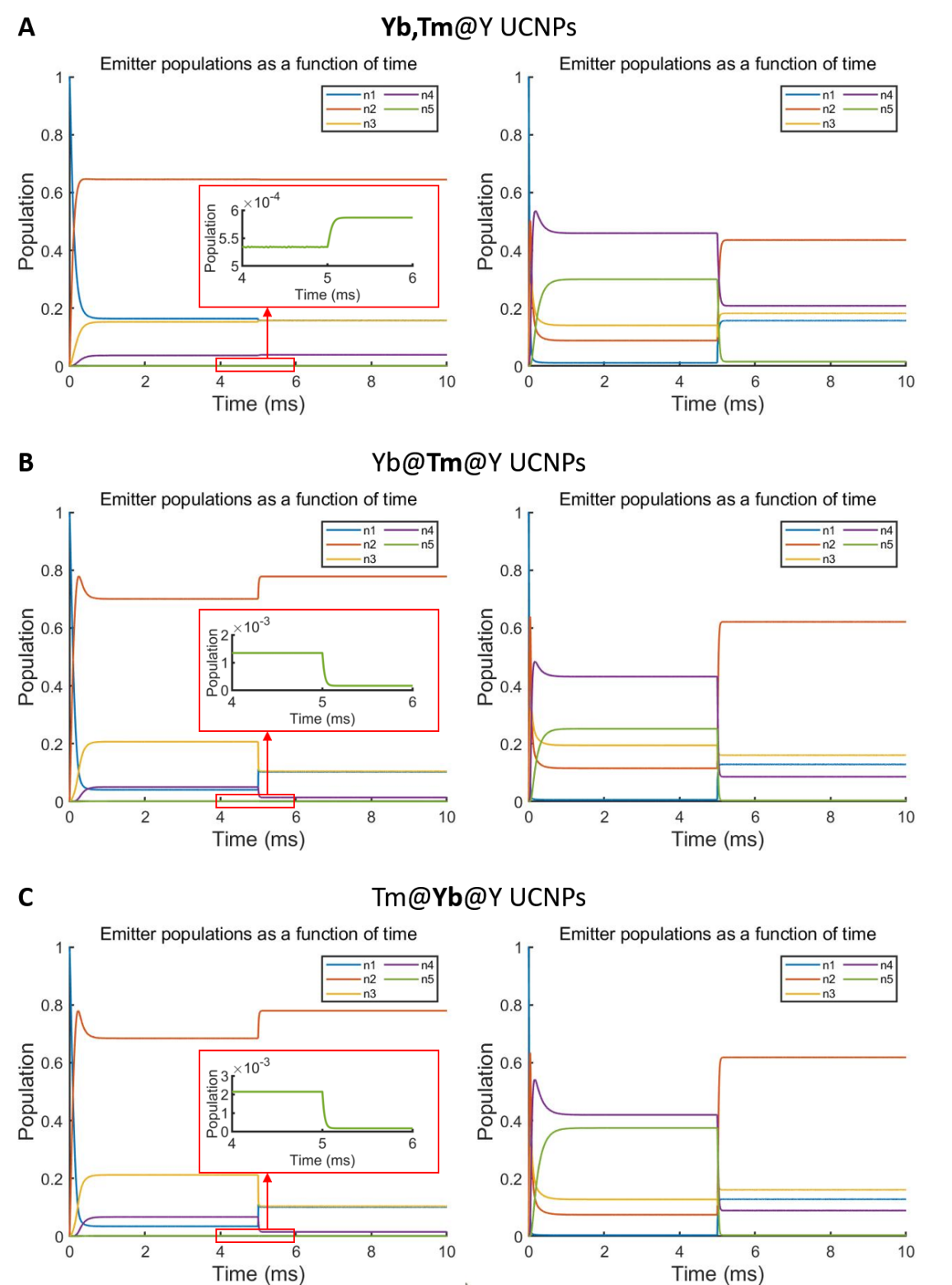


**Figure S8:** Theoretical modeling of the time-dependent electronic population distribution in core-shell UCNPs driven by topology-based ETNs under dual-beam irradiation, with a 980-nm excitation switched on at *t* = 0 ms and an 808-nm depletion switched on at *t* = 5 ms. The powers of the 980-nm excitation are of 0.1 mW (left column) and 1 mW (right column), and an 808-nm depletion at a power of 10 mW. **A**) Dynamics in **Yb,Tm**@Y UCNPs under dual-beam irradiation. Inset: magnified view of the distribution of level *n_5_*. **B**) Dynamics in Yb@**Tm**@Y UCNPs under dual-beam irradiation. Inset: magnified view of the distribution of level *n_5_*. **C**) Dynamics in Tm@**Yb**@Y UCNPs under dual-beam irradiation. Inset: magnified view of the distribution of level *n_5_*.


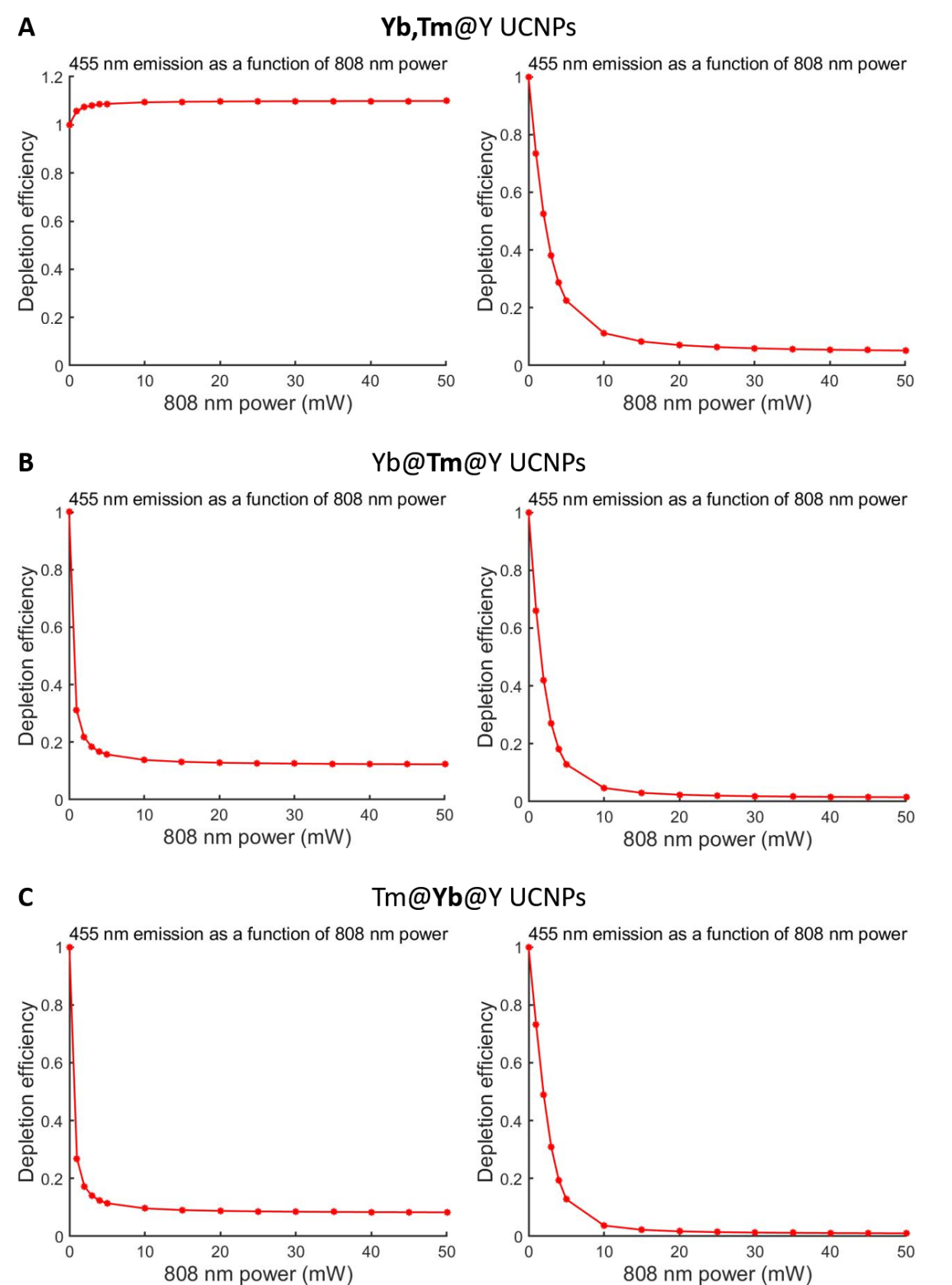


**Figure S9:** Theoretical modeling of the depletion efficiency in core-shell UCNPs driven by topology-based ETNs under dual-beam irradiation. **A**) Depletion efficiency in **Yb,Tm**@Y UCNPs under 980-nm excitation and an 808-nm depletion at increasing power. **B**) Depletion efficiency in Yb@**Tm**@Y UCNPs under 980-nm excitation and an 808-nm depletion at increasing power. **C**) Depletion efficiency in Tm@**Yb**@Y UCNPs under 980-nm excitation and an 808-nm depletion at increasing power. The powers of the 980-nm excitation are of 0.1 mW (left column) and 1 mW (right column).


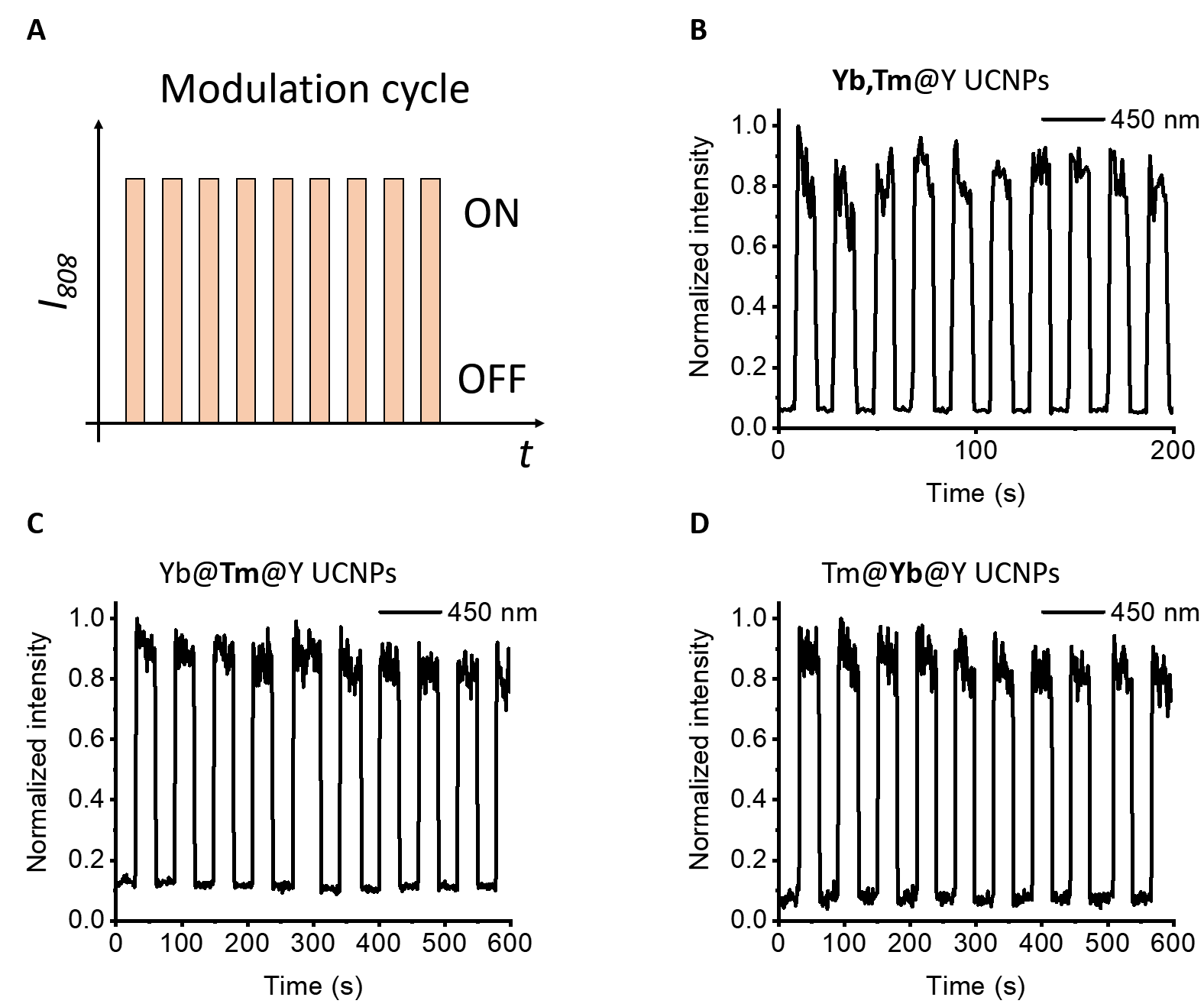


**Figure S10: A**) Schematic of the ‘ON’ and ‘OFF’ switching of the 808-nm depletion laser, where the laser is modulated through open/close cycles by an optical shutter. **B**) Normalized 450-nm UCL emission of **Yb,Tm**@Y UCNPs under 980-nm excitation at a power of 1 mW and 808-nm depletion at a power of 10 mW, with modulation cycles of 10 s of ‘ON’ switching and 10 s of ‘OFF’ switching. **C**) Normalized 450-nm UCL emission of Yb@**Tm**@Y UCNPs under 980-nm excitation at a power of 0.1 mW and 808-nm depletion at a power of 10 mW, with modulation cycles of 30 s of ‘ON’ switching and 30 s of ‘OFF’ switching. **D**) Normalized 450-nm UCL emission of Tm@**Yb**@Y UCNPs under 980-nm excitation at a power of 0.1 mW and 808-nm depletion at a power of 10 mW, with modulation cycles of 30 s of ‘ON’ switching and 30 s of ‘OFF’ switching for the 808-nm depletion laser.


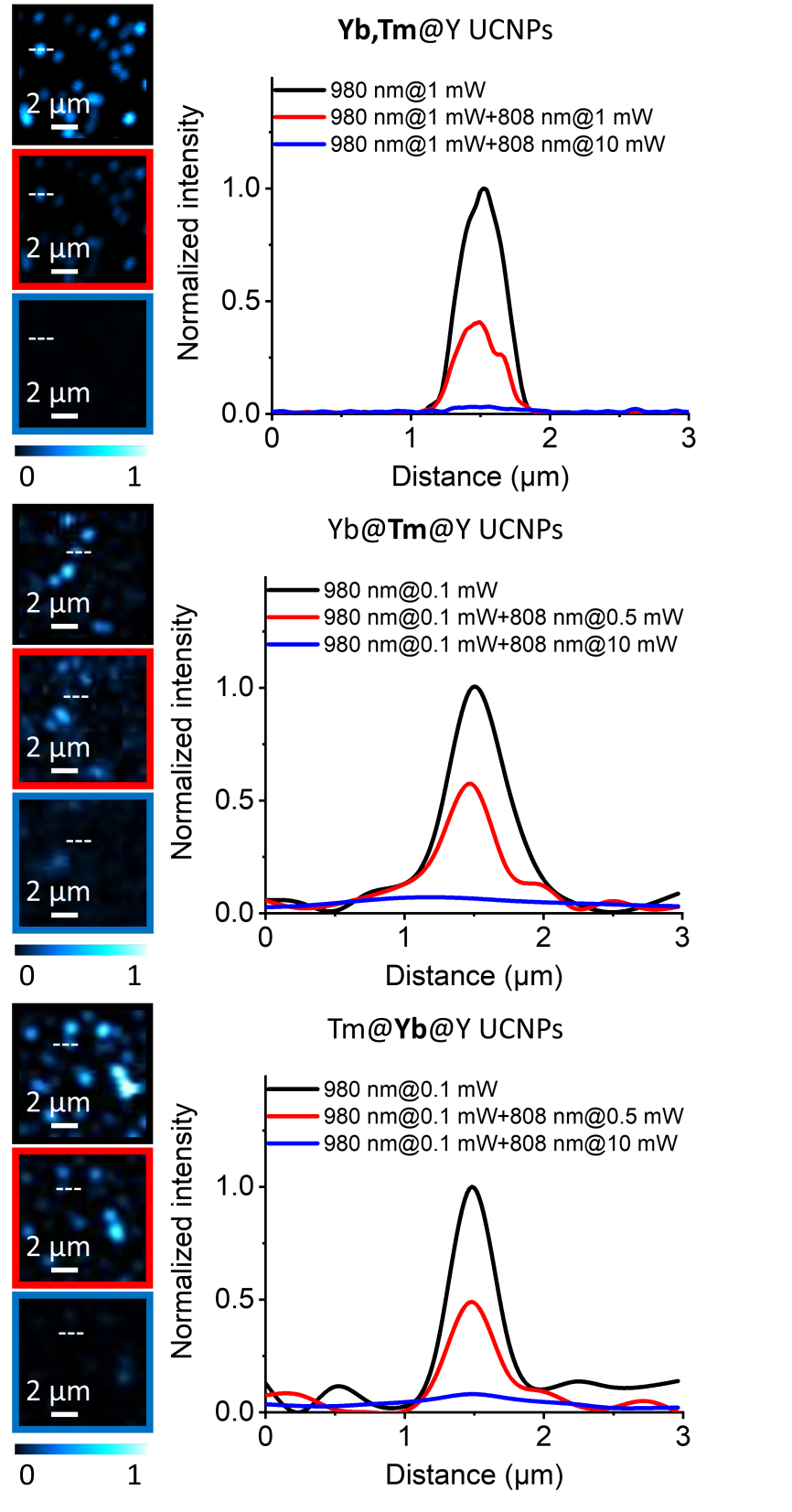


**Figure S11:** Confocal microscopy imaging by detecting the 450-nm UCL emission from the core-shell UCNPs (from top to bottom: **Yb,Tm**@Y, Yb@**Tm**@Y, and Tm@**Yb**@Y UCNPs) under 980-nm CW excitation and 808-nm CW depletion laser irradiation. The corresponding normalized intensity profile along the white dashed line is also shown. Scale bar: 2 μm.


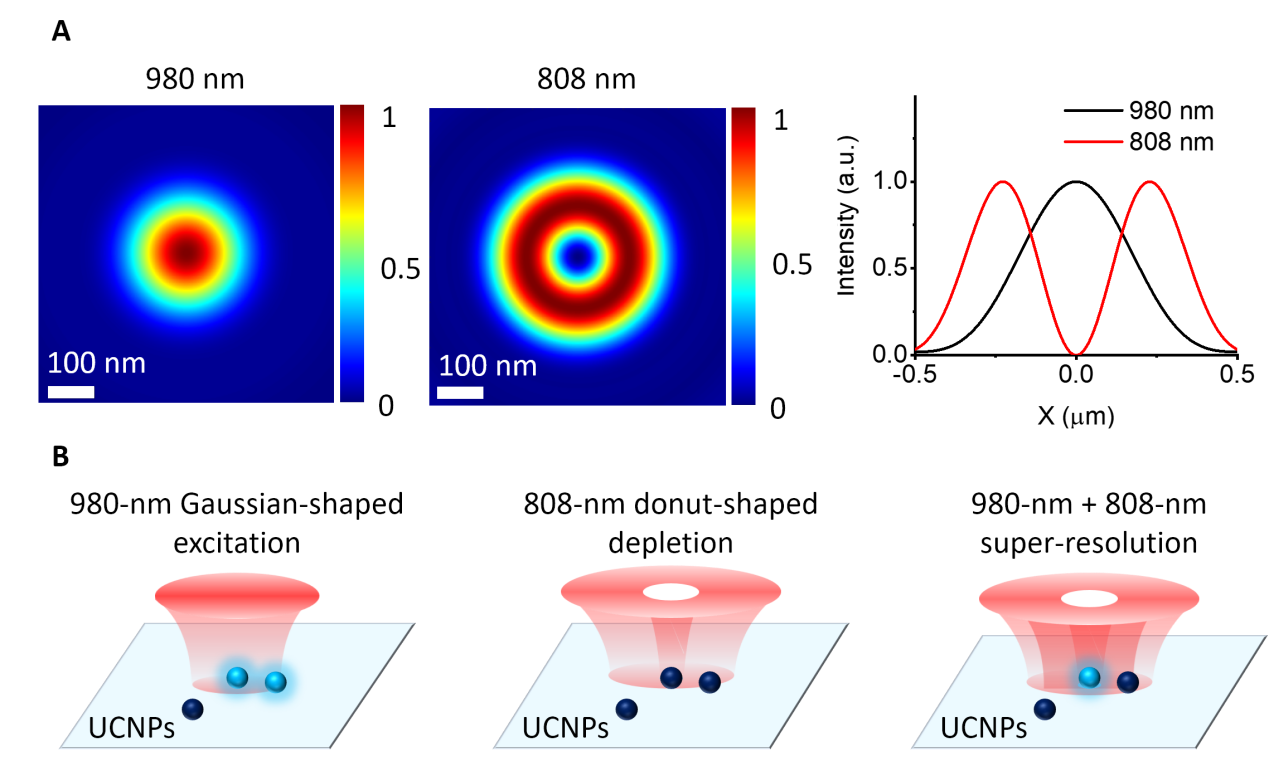


**Figure S12: A**) Simulated normalized intensity distribution of the 980-nm Gaussian-shaped excitation laser (left) and the 808-nm donut-shaped depletion laser (center) in the x-y plane of the focal region for stimulated emission depletion (STED)-based upconversion STED (U-STED) microscopy, accompanied by the normalized cross-section of their intensity profiles (right). Scale bar: 100 nm. **B**) Schematic illustration of U-STED microscopy imaging, utilizing a Gaussian excitation profile at 980 nm and a donut-shaped depletion profile at 808 nm in the far field.


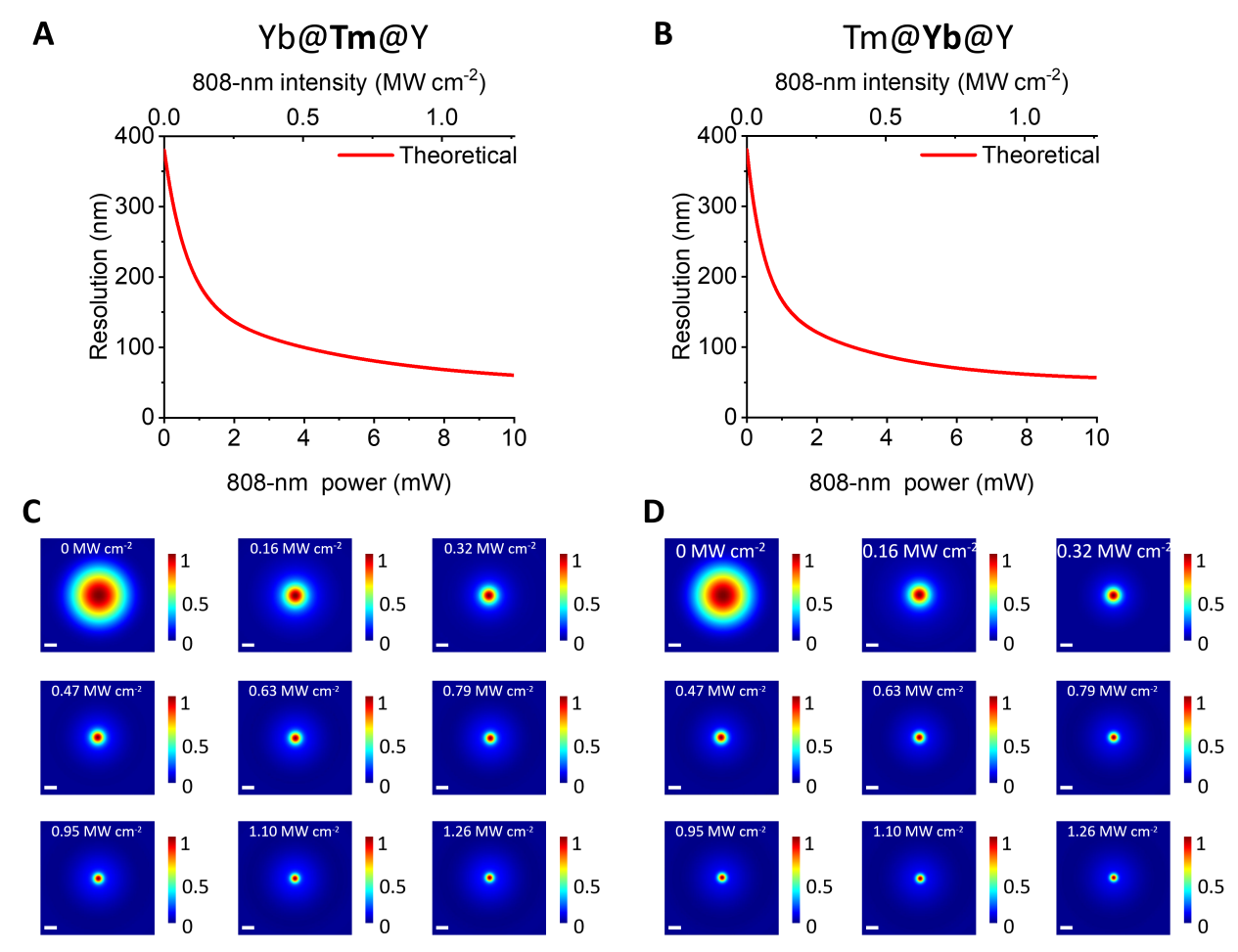


**Figure S13:** Theoretical modeling of resolution in U-STED microscopy using core-shell UCNPs under dual-beam super-resolution irradiation with a 980-nm Gaussian-shaped excitation laser at 0.03 MW cm^-^² and an 808-nm donut-shaped depletion laser. **A**) Resolution with Yb@**Tm**@Y UCNPs under increasing intensity of the 808-nm donut-shaped depletion laser. **B**) Resolution with Tm@**Yb**@Y UCNPs under increasing intensity of the 808-nm donut-shaped depletion laser. **C**) Simulated focal spot size in Yb@**Tm**@Y UCNPs under dual-beam super-resolution irradiation. Scale bar: 100 nm. **D**) Simulated focal spot size in Tm@**Yb**@Y UCNPs under dual-beam super-resolution irradiation. Scale bar: 100 nm.

**
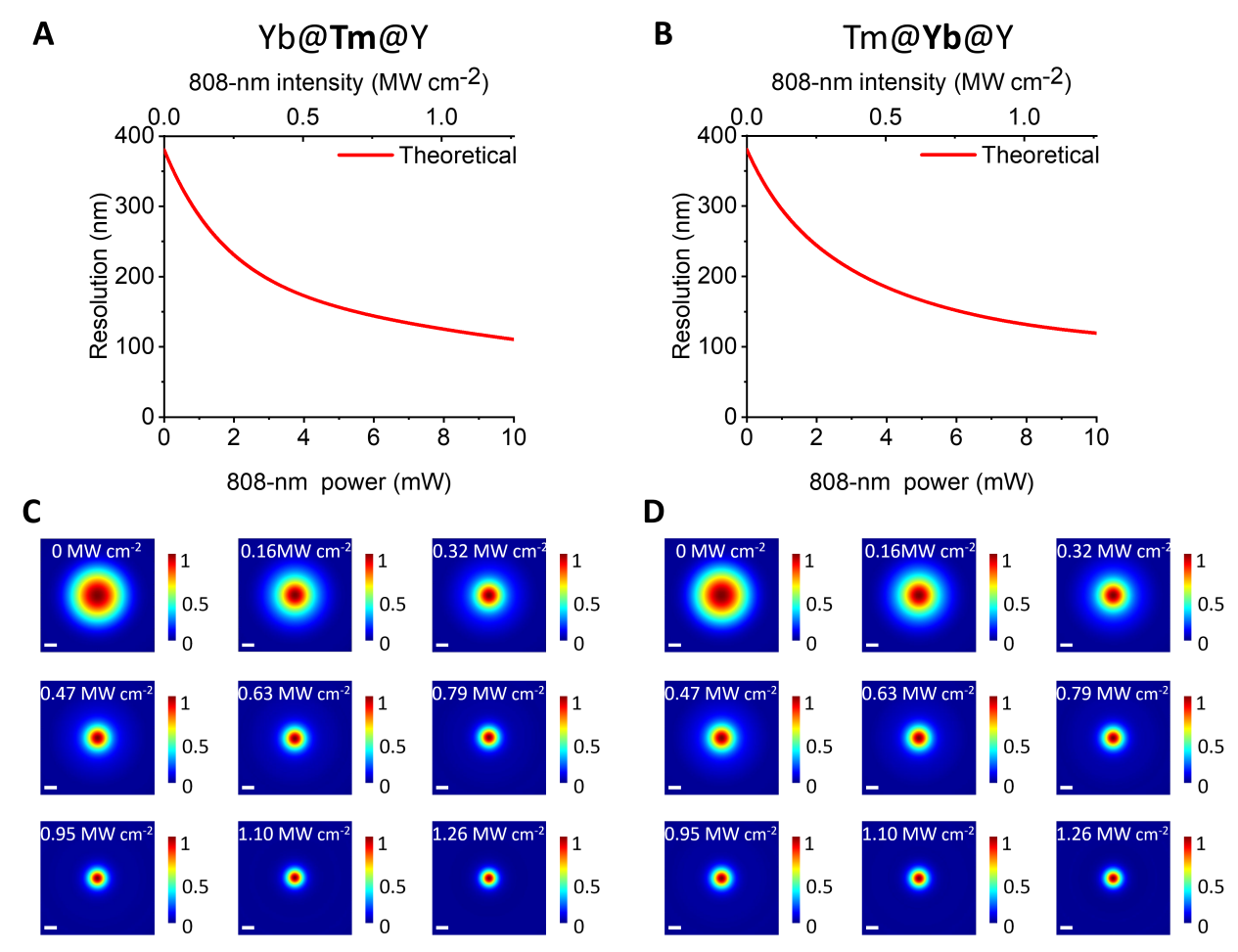
Figure S14:** Theoretical modeling of resolution in U-STED microscopy using core-shell UCNPs under dual-beam super-resolution irradiation with a 980-nm Gaussian-shaped excitation laser at 0.3 MW cm^-^² and an 808-nm donut-shaped depletion laser. **A**) Resolution with Yb@**Tm**@Y UCNPs under increasing intensity of the 808-nm donut-shaped depletion laser. **B**) Resolution with Tm@**Yb**@Y UCNPs under increasing intensity of the 808-nm donut-shaped depletion laser. **C**) Simulated focal spot size in Yb@**Tm**@Y UCNPs under dual-beam super-resolution irradiation. Scale bar: 100 nm. **D**) Simulated focal spot size in Tm@**Yb**@Y UCNPs under dual-beam super-resolution irradiation. Scale bar: 100 nm.


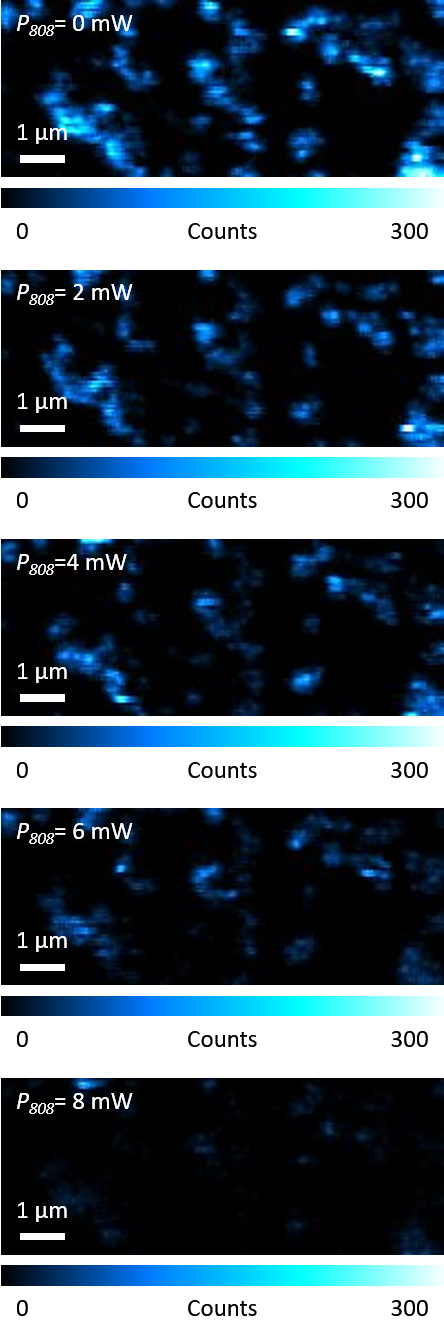


**Figure S15:** Confocal and U-STED microscopy using Yb@**Tm**@Y UCNPs under irradiation of a 980-nm Gaussian-shaped excitation laser at 0.03 MW cm^-2^, combined with an 808-nm donut-shaped depletion laser at progressively increasing powers. Pixel dwell time: 10 ms. Scale bar: 1 μm.

**Table S1:** Summary of the benchmark parameters of typical STED microscopy and U-STED microscopy.

|  | **Benchmark parameters** | | | | | | |
| --- | --- | --- | --- | --- | --- | --- | --- |
| **Technique** | **Probe** | **Dual-beam optical system** | ***λ_ex._* + *λ_de._* (nm)** | **Laser intensity (MW cm^-2^)** | ***λ_em._* (nm)** | **Lateral resolution (nm)** | **Pixel dwell time (μs pixel^-1^)** |
| Typical STED^1^ | Fluorophores | Pulsed | Visible | 1,000 - 10,000 | Visible | ~*λ_ex._*/12 | 20-200 |
| U-STED^2^ | NaYF_4_: 20%Yb^3+^, 8%Tm^3+^  (13 nm) | CW | 980 + 808 | 0.66 (ex.) + 7.5 (de.) | 455 | 28 (*λ_ex._*/36) | 4,000 |
| U-STED^3^ | NaYF_4_: 18%Yb^3+^, 10%Tm^3+^  (10 nm) | CW | 975 + 810 | N.A. | 455 | 66 (*λ_ex._*/15) | 4,000 |
| U-STED^4^ | NaYF_4_ @ NaYbF_4_: 10%Tm^3+^  (29 nm) | CW | 975 + 810 | N.A. | 455 | 72 (*λ_ex._*/14) | 10 |
| U-STED^5^ | NaYF_4_: 3%Nd^3+^  (17 nm) | CW | 740 + 1,064 | 0.08 (ex.) + 4.5 (de.) | 450 | 34 (*λ_ex._*/22) | 100 |
| U-STED^6^ | NaYF_4_: 20%Yb^3+^, 8%Tm^3+^  (39 nm) | CW | 980 + 808 | 0.27 (ex.) + 3.4 (de.) | 455 | 33 (*λ_ex._*/30) | 2,000 |
| U-STED [This work] | NaYF_4_: 8%Tm^3+^ @ NaYF_4_: 20%Yb^3+^ @ NaYF_4_  (52 nm) | CW | 980 + 808 | 0.03 (ex.) + 1 (de.) | 455 | 65 (*λ_ex._*/15) | 10,000 |

**Other benchmark parameters:**

- Field of view: similar to confocal 100×100 µm^2^ for typical STED and U-STED;

- Photon budget: limited for typical STED / infinite, non-bleaching for U-STED;

- Imaging depth: small for typical STED / large for U-STED;

- Photo-toxicity: high for typical STED / moderate for U-STED;

- Cost of light source: ~350,000 USD for 2 pulsed lasers for typical STED / ~6,000 USD for 2 CW lasers for U-STED.

**Table S2:** Calculated lanthanide ion quantity in core-shell UCNPs.

| **UCNP composition and structure** | **Yb^3+^ quantity** | **Tm^3+^ quantity** |
| --- | --- | --- |
| NaYF₄:25%Yb,5%Tm @ NaYF₄ | 5.22×10^4^ | 1.04×10^4^ |
| NaYF₄:25%Yb @ 5%Tm @ NaYF₄ | 3.93×10^4^ | 9.39×10^3^ |
| NaYF₄:5%Tm @ 25%Yb @ NaYF₄ | 4.80×10^4^ | 1.19×10^4^ |
| NaYF₄:**25%Yb, 5%Tm** @ NaYF₄ | 1.32×10^5^ | 2.65×10^4^ |
| NaYF₄:25%Yb @ **5%Tm** @ NaYF₄ | 1.08×10^5^ | 2.81×10^4^ |
| NaYF₄:5%Tm @ **25%Yb** @ NaYF₄ | 1.68×10^5^ | 2.92×10^4^ |

**Table S3:** Summary of the parameters used in the theoretical modeling of UCL emission in core-shell UCNPs driven by topology-based ETNs (from refs. 1, 7-9).

| For **Yb,Tm**@Y UCNPs | | | | | | | | | | | | | | | | | | | |
| --- | --- | --- | --- | --- | --- | --- | --- | --- | --- | --- | --- | --- | --- | --- | --- | --- | --- | --- | --- |
| **Absorption cross-section (cm^2^)** | | | | | |  | **Decay rate (s^-1^)** | | | | | | | | | | | | |
| *σ_Yd_* (cm^2^) | | | *σ_Tm_* (cm^2^) | | |  | *W_s_* | | *W_2_* | | *W_3_* | | | *W_4_* | | | *W_5_* | | |
| 2.5×10^-20^ | | | 7×10^-21^ | | |  | 8.2×10^3^ | | 6.2×10^3^ | | 1.5×10^4^ | | | 1.2×10^4^ | | | 3.2×10^3^ | | |
| **Upconversion coefficients (s^-1^)** | | | | | |  | **Energy migration (s^-1^)** | | | |  | **Cross-relaxation coefficients (s^-1^)** | | | | | | | |
| *c_1_* | *c_2_* | | *c_3_* | | *c_4_* |  | *EM* | | | |  | *k_31_* | | | *k_41_* | | | *k_51_* | |
| 6×10^4^ | 6×10^3^ | | 7×10^4^ | | 5×10^3^ |  | 1.2×10^2^ | | | |  | 1×10^5^ | | | 1.5×10^5^ | | | 2.5×10^5^ | |
| **Branching ratios** | | | | | | | | | | | | | | | | | | | |
| *b_31_* | | *b_32_* | | *b_41_* | | *b_42_* | | *b_43_* | | *b_51_* | | | *b_52_* | | | *b_53_* | | | *b_54_* |
| 0.27 | | 0.73 | | 0.18 | | 0.24 | | 0.58 | | 0.24 | | | 0.23 | | | 0.20 | | | 0.33 |
| For Yb@**Tm**@Y UCNPs | | | | | | | | | | | | | | | | | | | |
| **Absorption cross-section (cm^2^)** | | | | | |  | **Decay rate (s^-1^)** | | | | | | | | | | | | |
| *σ_y_* (cm^2^) | | | *σ_Tm_* (cm^2^) | | |  | *W_s_* | | *W_2_* | | *W_3_* | | | *W_4_* | | | *W_5_* | | |
| 2.5×10^-20^ | | | 7×10^-21^ | | |  | 8.2×10^3^ | | 5×10^3^ | | 1×10^4^ | | | 1.8×10^4^ | | | 3.4×10^3^ | | |
| **Upconversion coefficients (s^-1^)** | | | | | |  | **Energy migration (s^-1^)** | | | |  | **Cross-relaxation coefficients (s^-1^)** | | | | | | | |
| *c_1_* | *c_2_* | | *c_3_* | | *c_4_* |  | *EM* | | | |  | *k_31_* | | | *k_41_* | | | *k_51_* | |
| 6×10^4^ | 6×10^3^ | | 7×10^4^ | | 5×10^3^ |  | 5×10^1^ | | | |  | 3.5×10^5^ | | | 4.5×10^5^ | | | 5×10^5^ | |
| **Branching ratios** | | | | | | | | | | | | | | | | | | | |
| *b_31_* | | *b_32_* | | *b_41_* | | *b_42_* | | *b_43_* | | *b_51_* | | | *b_52_* | | | *b_53_* | | | *b_54_* |
| 0.27 | | 0.73 | | 0.18 | | 0.24 | | 0.58 | | 0.24 | | | 0.23 | | | 0.20 | | | 0.33 |
| For Tm@**Yb**@Y UCNPs | | | | | | | | | | | | | | | | | | | |
| **Absorption cross-section (cm^2^)** | | | | | |  | **Decay rate (s^-1^)** | | | | | | | | | | | | |
| *σ_yd_* (cm^2^) | | | *σ_Tm_* (cm^2^) | | |  | *W_s_* | | *W_2_* | | *W_3_* | | | *W_4_* | | | *W_5_* | | |
| 2.5×10^-20^ | | | 7×10^-21^ | | |  | 8.2×10^3^ | | 4.5×10^3^ | | 1.3×10^4^ | | | 1.2×10^4^ | | | 2.5×10^3^ | | |
| **Upconversion coefficients (s^-1^)** | | | | | |  | **Energy migration (s^-1^)** | | | |  | **Cross-relaxation coefficients (s^-1^)** | | | | | | | |
| *c_1_* | *c_2_* | | *c_3_* | | *c_4_* |  | *EM* | | | |  | *k_31_* | | | *k_41_* | | | *k_51_* | |
| 6×10^4^ | 6×10^3^ | | 7×10^4^ | | 5×10^3^ |  | 3.2×10^2^ | | | |  | 3.2×10^5^ | | | 4.8×10^5^ | | | 5.2×10^5^ | |
| **Branching ratios** | | | | | | | | | | | | | | | | | | | |
| *b_31_* | | *b_32_* | | *b_41_* | | *b_42_* | | *b_43_* | | *b_51_* | | | *b_52_* | | | *b_53_* | | | *b_54_* |
| 0.27 | | 0.73 | | 0.18 | | 0.24 | | 0.58 | | 0.24 | | | 0.23 | | | 0.20 | | | 0.33 |

**References:**

1. Klar, T. A. et al. Fluorescence microscopy with diffraction resolution barrier broken by stimulated emission. *Proceedings of the National Academy of Sciences of the United States of America* **97**, 8206-8210, doi: 10.1073/pnas.97.15.8206 (2000).
2. Liu, Y. et al. Amplified stimulated emission in upconversion nanoparticles for super-resolution nanoscopy. *Nature* **543**, 229-233, doi: 10.1038/nature21366 (2017).
3. Zhan, Q. et al. Achieving high-efficiency emission depletion nanoscopy by employing cross relaxation in upconversion nanoparticles. *Nature Communications* **8**, 1058, doi: 10.1038/s41467-017-01141-y (2017).
4. Peng, X. et al. Fast upconversion super-resolution microscopy with 10 μs per pixel dwell times. *Nanoscale* **11**, 1563-1569, doi: 10.1039/C8NR08986H (2019).
5. Guo, X. et al. Achieving low-power single-wavelength-pair nanoscopy with NIR-II continuous-wave laser for multi-chromatic probes. *Nature Communications* **13**, 2843, doi: 10.1038/s41467-022-30114-z (2022).
6. Liu, Y. et al. Population control of upconversion energy transfer for stimulation emission depletion nanoscopy. *Advanced Science* **10**, e2205990, doi: 10.1002/advs.202205990 (2023).
7. Wang, Y. F. et al. Nd^3+^-sensitized upconversion nanophosphors: efficient in vivo bioimaging probes with minimized heating effect. *ACS Nano* **7**, 7200-7206, doi: 10.1021/nn402601d (2013).
8. Smith, A. V. et al. Mode instability thresholds for Tm-doped fiber amplifiers pumped at 790 nm. *Optics Express* **24**, 975-992, doi: 10.1364/OE.24.000975 (2016).
9. Villanueva-Delgado, P. et al. Judd-Ofelt analysis of β-NaGdF_4_: Yb^3+^, Tm^3+^ and β-NaGdF_4_:Er^3+^ single crystals. *Journal of Luminescence* **189**, 84-90, doi: 10.1016/j.jlumin.2016.04.023 (2017).
